# Movement Mechanisms Harness Lévy Flight for Energy‐Efficient Wastewater Treatment in Microalgae–Bacteria Systems

**DOI:** 10.1002/advs.202504676

**Published:** 2025-09-08

**Authors:** Luyu Zhang, Yu Tian, Lipin Li, Wei Zhan, Huihang Sun, Nanqi Ren, Zhurui Tang, Huu Hao Ngo

**Affiliations:** ^1^ State Key Laboratory of Urban Water Resource and Environment (SKLUWRE) School of Environment Harbin Institute of Technology Harbin 150090 China; ^2^ College of Agronomy and Biotechnology Southwest University Beibei Chongqing 400715 China; ^3^ Faculty of Engineering University of Technology Sydney P.O. Box 123, Broadway Sydney NSW 2007 Australia

**Keywords:** cellular movement model, energy‐efficient wastewater treatment, Lévy Flights behavior, microalgae–bacteria symbiosis, self‐aggregation mechanisms

## Abstract

Microalgae–bacteria symbiosis system is significant for sustainable and low‐carbon wastewater treatment, with self‐aggregation being key to its stable operation and effective pollutant removal. Cellular motility is the main driving force behind self‐aggregation, crucial for symbiosis stability, but the characteristics and patterns involved still remain largely unexplored. Here, cellular movement dynamics into the microalgae‐activated sludge model (ASM3) is incorporated, enabling synchronized simulation of metabolic activities and movement behaviors through physical and biochemical interactions in bioreactor systems. These findings indicate that microalgae induce bacterial movement towards Lévy flights, thereby increasing the bacterial encounter rate by 12.20%, augmenting signaling molecule concentration and biomass by 20.0% and 27.3%, respectively, which in turn strengthens the bacteria self‐aggregation effect. Through practical reactor operations with metagenomic analysis, the efficacy of this model in elucidating self‐aggregation is further corroborated, improving system stability and pollutant removal efficiency. An optimized microalgae–bacteria system reduces energy costs associated with cellular aggregation processes, economizing on the cost of chemotaxis‐related proteins. This study not only elucidate the unique role of Lévy flight in self‐aggregation, enhancing the understanding of microalgae–bacteria symbiosis, but also establish response mechanisms between motility patterns and operation dynamics. This allows for targeted regulation across various biosystems, ensuring cost‐effective wastewater treatment and proactive prediction.

## Graphical abstract

Here, our novel model that integrates metabolism with motility dynamics reveals that microalgae shift bacterial movement toward Lévy flight patterns, enhancing encounter rates and self‐aggregation. This microecological manipulation reduces energy costs, strengthens functions such as pollutant removal and system stability. It provides a new pathway for optimizing wastewater treatment through identifying and modulating motility patterns.

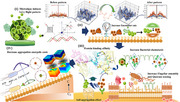

## 1 Introduction

Microalgae and bacteria can create a complex symbiotic microenvironment through collaborative metabolism. Specifically, microalgae provide the oxygen and organic compounds for bacterial growth, while bacteria supply carbon dioxide to enhance the photosynthetic efficiency of microalgae, fostering a symbiotic relationship. This interaction not only enhances system stability and pollutant removal in wastewater treatment but also facilitates biomass energy recovery, making it a promising wastewater treatment technology.^[^ ^1^, ^2^, ^3^, ^4^, ^5^ ^]^ However, challenges like the loss and fixation difficulty of microalgae significantly hinder the large‐scale application and development of the process.^[^ ^6^, ^7^ ^]^ Through self‐aggregation, microalgae–bacteria granular sludge (ABGS) system can be formed, establishing and maintaining long‐term stable symbiosis. ABGS enhances microalgae–bacteria interaction, improves settling performance, and increases pollutant removal efficiency, making it a potent method for developing wastewater treatment processes with long‐term stability and high pollutant removal efficiency.^[^ ^1^, ^8^, ^9^, ^10^, ^11^ ^]^

Cellular motility is the core dynamic driving self‐aggregation, regulated by quorum sensing.^[^ ^12^, ^13^, ^14^, ^15^, ^16^ ^]^ It influences microalgae–bacteria interaction, alters metabolic processes, and promotes the symbiotic microenvironment formation. Cellular motility is a multifaceted and intricate phenomenon subject to the collective implications of fluid dynamics, chemical signaling, and surface interactions.^[^ ^17^, ^18^, ^19^, ^20^ ^]^ Such factors act in concert to drive cells to incessantly adjust their movement patterns,^[^ ^21^, ^22^ ^]^ aligning movement conducive to chemotactic gradients (substrates, signaling molecules, etc.), thereby intensifying chemotaxis and potentiating towards strategic localization of substrates and other environmental resources.^[^ ^23^, ^24^, ^25^, ^26^ ^]^ For instance, in habitats characterized by heterogeneous substrate distributions, bacterial movement can adapt to significantly expand the spatial range for substrate search.^[^ ^27^ ^]^ Although cellular motility plays a pivotal role in self‐aggregation and the symbiosis between microalgae and bacteria, it is influenced by a multitude of mechanisms and environmental factors.^[^ ^20^, ^28^ ^]^ Moreover, the inherent complexity of biological systems making that isolating cells for motility studies can alter the biological state of the system, leaving the motility characteristics and specific mechanisms through traditional experimental methods in the symbiotic process largely unexplored.

Researchers have developed theoretical models simulating complex cellular movements from various perspectives to address these challenges, providing avenues for investigating bacterial cell movement characteristics. For example, cell kinetics dynamic models simulate bacterial cell movements under physical influences such as fluid vorticity and spatial hindrance;^[^ ^17^, ^19^, ^29^, ^30^ ^]^ jump‐diffusion models simulate cell movements under far‐field flow effects and near‐field interactions.^[^ ^31^, ^32^ ^]^ Additionally, troves of chemotaxis models lay out chemokinetically governed cellular movement traits that vary under the influence of biochemical signal gradients.^[^ ^18^, ^25^, ^33^ ^]^ While existing motility models comprehensively characterize cellular movement, they have not yet been applied to wastewater treatment systems. Among the ASM series models, ASM3 was widely used due to its consideration of storage processes as an important metabolic intermediate and consistent description of endogenous respiration. The model encapsulates biological dynamic processes with attention to biomass growth and substrate consumption,^[^ ^34^, ^35^, ^36^ ^]^ with key characteristics include clear categorization of microbial populations and substrates, process‐based description of growth, decay and substrate utilization, stoichiometric and kinetic parameter standardization. But it cannot directly explore cell motility. Thus, by integrating microbial motility with bioreactor kinetics through metabolic‐movement coupling mechanisms, we simulated cellular movement in wastewater treatment systems. These coupling mechanisms combine physical forces and biochemical effects with cellular metabolic processes through mutual interaction mechanisms and unified time‐step iterations, creating an integrated metabolic‐movement coupled model in wastewater treatment systems. Our study elucidates the characteristics and patterns of cell motility in wastewater treatment, providing deeper insights into the mechanisms underlying self‐aggregation and the intricate symbiosis between microalgae and bacteria.

Here, to our knowledge, for the first time, we incorporated cell motility dynamics into the ASM3‐microalgae model through the iDynoMiCS framework to simulate bacterial and microalgal movement under biodynamic processes. The model was calibrated through sensitivity analysis and extensive foundational experiments. Integration of experimental observations with metagenomic analysis provides insights into microalgal effects on bacterial movement and movement‐driven operational mechanisms in bioreactor systems. This approach elucidates cellular movement patterns and traits, which are pivotal for self‐aggregation in symbiosis processes, and further advances energy‐efficient ecological regulation at the microscale. The primary research objectives include 1) Investigating the dynamic motility characteristics of bacterial cells during self‐aggregation, including their trajectories, modes of movement, and velocity distribution; 2) Exploring the self‐aggregation behavior and encounter rate of bacterial cells under these motility characteristics; and simulating the distribution of signaling molecules and biomass concentration at system equilibrium; 3) Exploring the impacts of movement characteristics on self‐aggregation processes and pollutant removal performance in bioreactor operations, in conjunction with metagenomic analysis to reveal driving mechanisms. This study establishes an integrated framework to investigate cellular motility characteristics in microalgae–bacterial systems, revealing critical mechanisms in self‐aggregation and symbiosis processes. The findings provide novel insights into microalgal‐bacterial interactions in wastewater treatment, contributing to energy‐efficient and sustainable treatment strategies at the microscopic scale.

## 2 Results

### 2.1 Microalgae Induce Shifts in Bacteria Movement

**Figure 1:**
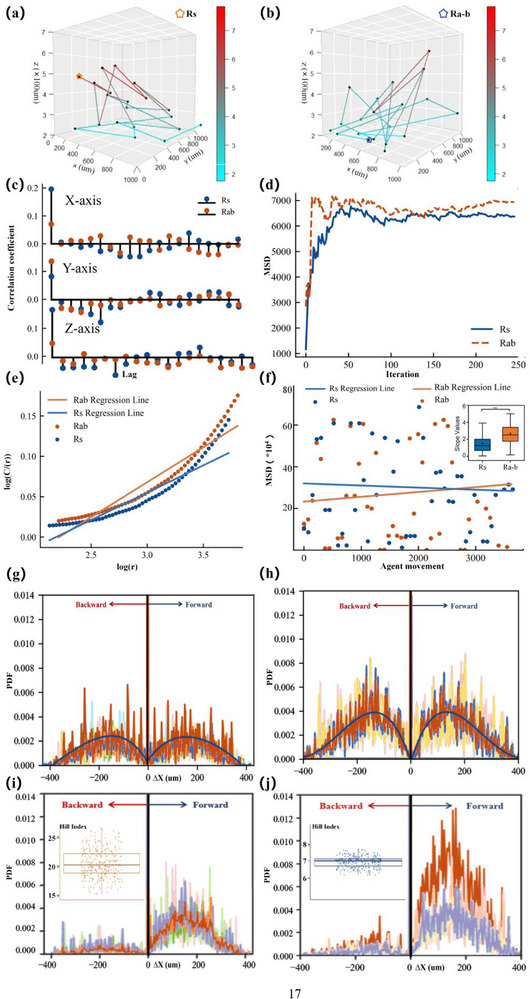
a,b) 3D simulation of bacterial cell trajectories in the two systems. The star symbols mark the starting points of each trajectory. The color bar indicates the height along the Z‐axis (in µm), with cooler colors (blue) representing lower positions and warmer colors (red) representing higher positions. Complete trajectory maps are provided in Figure S3 (Supporting Information). c) Autocorrelation function, with the results suggesting less autocorrelation in the microalgae–bacteria system compared to the bacteria system across the three axes, implying a greater degree of randomness in the cell movements of the microalgae–bacteria system. d) Degree of aggregation (lower MSD indicates higher degree of aggregation). e) Correlation dimension (higher values and a steeper gradient denoting enhanced randomness). f) Diffusion index (a steeper slope of the fitted line indicates a rapid diffusion rate). g,i) Displacement probability density function of the Rs system and the Ra‐b system, depicting a simulation without controlled directionality in three dimensions. h,j) Displacement probability density function of Rs system and Ra‐b system in a directed simulation to magnify differences; the inset in the top left corner is a Hill index plot, representing the tail index.

Based on 70 d simulations with the validated model (see Figure S10, Supporting Information for Model validity and accuracy analysis), microalgae in the Ra‐b system significantly modified bacterial trajectories, enhancing spatial exploration and increasing the vigor of cellular displacement (**Figure** **1**a,b and Figures S3 and S4, Supporting Information). Subsequently, we calculated the expected speed integral based on the displacement probability density function (PDF), indicating average velocities of 313.08 µm min^−1^ for the Ra‐b system compared to 188.28 µm min^−1^ for the Rs system (Figure 1g). These findings highlight a marked increase in bacterial cell movement and displacement within the microalgae–bacteria system, characterized by more considerable leap consistent with Lévy flight pattern.^[^ ^24^, ^37^ ^]^ Notably, compared with the Rs system, the PDF for the Ra‐b system exhibited a heavier‐tailed distribution. Specifically, the tail index of the displacement distribution of the microalgae–bacteria system is 6.96, lower than that of 20.61 observed in the bacteria system (Figure 1h,j), a characteristic of heavier‐tailed distribution that is fundamental for conformity with the Lévy flight pattern.^[^ ^37^ ^]^ Lévy flight is a superdiffusive random walk pattern prevalent in biological systems, such as animal foraging strategies, and physical systems, such as quantum distribution characteristics.^[^ ^38^, ^39^, ^40^, ^41^ ^]^ In biological systems, when searchers (predators, pollinators, etc.) are larger or faster, Lévy flight can enhance the encounter rate,^[^ ^42^ ^]^ which is also foundational for bacterial interactions.^[^ ^43^ ^]^

Trajectory analyses employing autocorrelation functions, correlation dimensions, and diffusion indices reveal that the bacteria cell motility pattern of Ra‐b was characterized by heightened randomness and nonlinearity in their temporal and spatial distribution, alongside an increase in diffusion dynamics‐hallmarks indicative of Lévy flight behavior (Figure 1c,e,f).^[^ ^44^, ^45^, ^46^ ^]^ The Ra‐b system exhibits autocorrelation values that are substantially reduced across all three axes in comparison to the Rs system, with the most dramatic decrements observed on the Z‐axis at 32.80%, followed by 23.50% and 18.98% on the Y and X axes, respectively, indicating a rise in stochasticity among spatial displacements.^[^ ^44^ ^]^ The inverse relationship between the slope of the correlation dimension and the spatial complexity of the system is evident. Our results showed a 27.99% increase in the slope for the Ra‐b system compared to the Rs system (Figure 1e), indicating a trajectory replete with enhanced complexity and dynamic nonlinearity.^[^ ^47^, ^48^, ^49^ ^]^ The diffusion index further substantiated these observations through Mean Square Displacement (MSD) analysis, which quantifies the spatial exploration patterns over time. The MSD slope (*α*) for the Ra‐b system was 2.32 times that of the Rs system, indicating broader diffusion range for cell movement^[^ ^45^, ^46^ ^]^ (Figure 1f). This significantly higher diffusion coefficient demonstrates a broader spatial exploration range and more efficient movement pattern in the Ra‐b system compared to the Rs system, indicating enhanced diffusive behavior consistent with Lévy flight characteristics. The observed dispersion of data points is attributed to the characteristic bacterial movement pattern that combines both short‐range and long‐range motions. The steeper regression line for the Ra‐b system indicates faster diffusion rates compared to the Rs system, consistent with our other analytical results (autocorrelation functions and correlation dimensions shown in Figure 1c,e). Statistical analysis of MSD slopes of diffusion index results from 200 independent simulations, followed by significance testing, confirmed that the absolute slope value of the Ra‐b system was significantly higher than that of the Rs system (*p* = 6.90e‐8), indicative of a broader diffusion range for cell movement^[^ ^45^, ^46^ ^]^(Figure 1f).

Our integrated model results demonstrate that microalgae act as an activator to alter the motility patterns of bacterial cells, leading to enhanced exploratory behavior, increased randomness and diffusion in movement, and a more pronounced presence of heavy‐tailed distributions. These observations align with the quintessential attributes of Lévy flight behavior, suggesting a potential shift in bacterial motility towards Lévy flight tendency. Lévy flight is characterized by a specific random walk pattern combining many small steps with occasional longer jumps, resulting in a power‐law distribution of step lengths. Its key defining features include: heavy‐tailed probability distributions, enhanced diffusive behavior, and increased spatial exploration with random jumps.^[^ ^24^, ^37^ ^]^

### 2.2 Lévy Flight Inclination Induced by Microalgae Facilitates Self‐Aggregation

**Figure 2:**
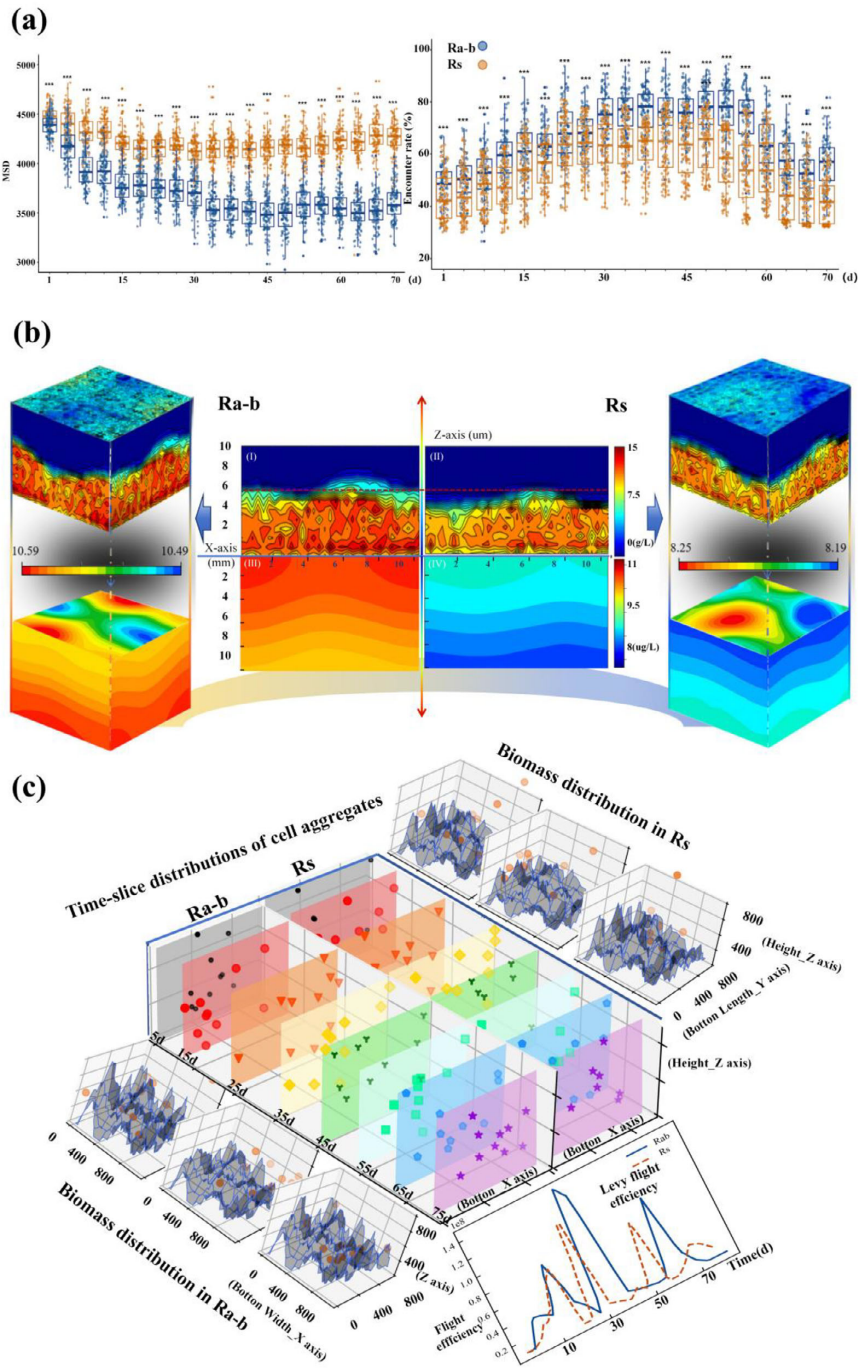
a) System aggregation degree and encounter rate, based on 200 simulations wherein 500 bacterial cells were randomly sampled at corresponding time points. Cohesiveness and aggregation effect were gauged using MSD as a metric, where higher MSD values indicate greater cell dispersion and lower aggregation effects. Cohesiveness was gauged using MSD as a metric. b) In a state of system equilibrium, 3D simulations of the system's biomass (I, II) and signaling molecule concentration (III, IV). The columnar bands atop signal molecules delineate distinct concentration intervals between the two systems. c) Spatiotemporal dynamics and movement efficiency analysis of bacterial cell distribution in in Ra‐b and Rs systems: temporal snapshots of cellular aggregation patterns across reactor cross‐sections (center panels), 3D biomass distribution at early, middle, and late operational stages (side panels), and Lévy flight efficiency throughout the operational cycle (bottom‐right panel).

To explore the influence of microalgae‐driven alterations in the Lévy flight pattern on the self‐aggregation while mitigating the potential impact of model‐inherent randomness, we executed 200 times simulations. The diffusion and aggregation degree of bacterial cells at different time points were characterized using MSD, where higher MSD values indicate greater spatial dispersion and weaker aggregation, while lower values represent stronger cell clustering and enhanced cohesiveness. As shown in **Figure** **2**a (left), the addition of microalgae significantly reduced bacterial MSD values, indicating enhanced cell aggregation and improved cohesiveness. Trajectory analyses further revealed that this enhanced bacterial aggregation resulted in an elevated encounter rate in the Ra‐b system (Figure 2a right), demonstrating a higher probability of encounter rate under the microalgae‐influenced Lévy flight pattern tendency. The correlation between reduced MSD (stronger aggregation) and increased encounter rate validates the effectiveness of microalgae in promoting bacterial interactions. Notably, the bacteria encounter rate in the Ra‐b system was 65.87%, surpassing the bacteria system by 12.20%, signifying an enhanced propensity for cellular interaction.^[^ ^43^ ^]^

The biomass distribution indicates cellular spatial arrangement and growth in the system, while the concentration of signaling molecules reflects cellular motility and quorum‐sensing capabilities.^[^ ^25^ ^]^ Accordingly, distribution simulations for biomass and signaling molecule concentrations were performed upon the systems in a steady state. Figure 2b indicated both biomass concentration and growth effects were amplified in the Ra‐b system, characterized by pronounced z‐axis stratification, suggesting a higher biomass density in the core. Complementarily, the distribution of signaling molecule, specifically N‐acyl homoserine lactones (AHLs) as the quorum sensing (QS) signal molecules in our study. Signal molecules concentration exhibited heightened density gradients, governed by common chemical factors shared by bacteria and microalgae in the system,^[^ ^25^ ^]^ reflecting the capacity of bacteria to sense concentration gradients,^[^ ^50^ ^]^ an observation corroborated by the efficiency analysis of Lévy flight patterns presented in Figure 2b,c. Thus, it is denoted that the microalgae‐induced alteration in motility patterns modifies cellular growth states and motile activity and enhances the overall system biomass and substrate sensitivity.

Furthermore, temporal cross‐sections that delineated multiple cells' locational dynamics during the cycle, providing a projection visualization of multi‐cellular dynamic motility. Figure 2c also dynamically represented biomass distribution. The results demonstrated that multi‐cellular movement and aggregation incidence were markedly elevated in the Ra‐b system, characterized by Lévy flight tendency. In comparison to the bacteria system, there was a statistically significant increase in bacterial cell aggregation by an average of 15.94% in the microalgae–bacteria system, with the 45th day manifesting a peak differential of 31.59% (Figure 2a,c). Overall, the simulation cycle suggested a progressive intensification of bacterial self‐aggregation that plateaued upon reaching a state of equilibrium. Intriguingly, despite the diminished aggregation in the trajectories of individual bacterial movements in Ra‐b system (Figure 1d), a higher degree of cellular aggregation was observed (Figure 2a,c). It is suspected that while microalgae influence bacterial cell trajectories to become more random, the resultant more considerable step lengths may facilitate closer spatial proximity among cells, leading to self‐aggregation.

In addition, we simulated integrating both bacterial and microalgae cells in the Ra‐b system to explore interspecific self‐aggregation (Figure S5, Supporting Information). Initial dispersion gave way to emergent combinations of bacteria‐bacteria and bacteria‐microalgae over time. Bacterial cells predominantly congregated centrally, with their proximity to microalgae gradually decreasing, significantly enhancing aggregation.

In summary, further simulation analysis revealed that microalgae‐induced Lévy flight behavioral patterns substantially elevated the likelihood of cellular interactions and enhanced biomass accretion. It increased the secretion of signaling molecules, thereby potentiating the self‐aggregation process. These interrelated factors collectively indicated that Lévy flight induced by microalgae was instrumental in promoting bacteria self‐aggregation by enhancing encounter rates, quintessential for bacterial interaction.^[^ ^42^, ^43^ ^]^ Extensive literature corroborates that self‐aggregation enhances system stability and pollutant remediation efficacy.^[^ ^1^, ^4^, ^5^, ^11^, ^51^ ^]^

### 2.3 Benefits of Microalgae‐Induced Pattern in Practical Reactor Operations

**Figure 3:**
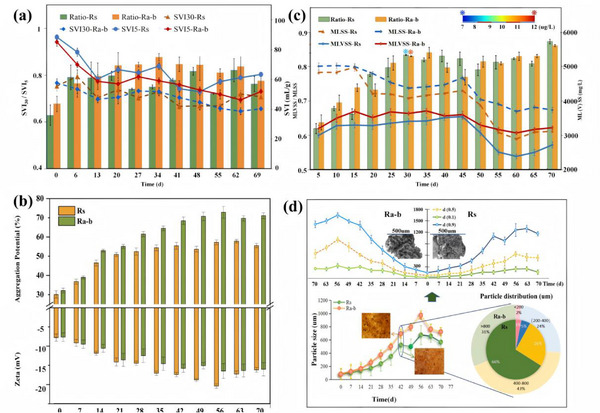
a) Sludge volume index (SVI) and settleable volume (SV) measurements. b) The aggregation capacity and zeta potential analysis of the Rs and Ra‐b systems. c) Mixed liquor suspended solids (MLSS) and mixed liquor volatile suspended solids (MLVSS) for the Rs and Ra‐b systems. d) Analysis of particle size and aggregate morphology, including changes in d 10%, d 50%, and d 90% at different time points, as well as the variation in the mean particle size of the systems.

We conducted bioreactor operation experiments, coupled with metagenomic analysis, to explore the impact of cellular motility on self‐aggregation and pollutant removal mechanisms. Practical operations confirmed that microalgae significantly contribute to the increase of biomass and signaling molecule concentrations as simulated by the models. At the end of the operational cycle, the MLSS of the microalgae–bacteria and bacteria systems were recorded at 3728 and 3104 mg L^−1^, respectively, with a significant rise in the MLVSS to MLSS ratio. Signal molecule analysis revealed that the Ra‐b system achieved significantly higher AHL concentrations (11.36 µg L^−1^) compared to the Rs system (8.27 µg L^−1^) at day 30, representing a 37% enhancement (**Figure** **3**c). Comprehensive temporal analysis across the full 70 d operational period confirmed this pattern, demonstrating that the Ra‐b system maintained consistently elevated signal molecule concentrations with 25–30% higher values throughout operation, particularly during the active aggregation phases (Figure S6, Supporting Information). Peak value in Ra‐b system of 13.73 µg L^−1^ was observed at day 40, compared to maximum concentrations of 10.58 µg L^−1^ in the Rs system. These findings are consistent with simulation results that microalgae can augment bacterial cells' perception and sensitivity to substrate concentrations, further amplifying the increase in biomass and signaling molecule concentrations (padj < 0.05).

Reactor operation metrics indicated an increased propensity for cellular self‐aggregation in microalgae–bacteria system, with a 52.93% aggregation potential (Ra‐b) at day 10, surpassing the 46.72% of bacteria system (*R*s). This disparity in aggregation potential progressively widened, peaking at day 49 with values of 70.72% (Ra‐b) versus 53.62% (Rs) (Figure 3b), signifying microalgae perpetuated bacterial aggregation potential through the whole operational cycle and further markedly enhanced granulation processes. Granulation completion standards are indicated by a d (0.1) value greater than 200 µm and a settling level SVI30/SVI5 ratio above 0.8.^[^ ^52^ ^]^ Figure 3a,d showed that the microalgae–bacteria system completed granulation around day 28, whereas bacteria system required approximately 49 d, achieving granulation 21 d earlier.

Experimental operation results also substantiated the role of microalgae in enhancing substrate utilization, pollutant removal, and system stability. In practical operation, microalgae facilitated the removal of carbon and nitrogen pollutants by enhancing substrate utilization. Compared to the bacteria system, the microalgae–bacteria system exhibited superior and stable removal efficiencies for carbon and nitrogen, with ammonia removal rate nearing completion and a total nitrogen removal rate of about 80%, marking a 16.7% increase over the bacteria system (Figure S9, Supporting Information). This enhanced nitrogen removal efficiency can be attributed to both direct assimilation by microalgae and improved bacterial removal efficiency through the Lévy flight movement patterns, which increased self‐aggregation and led to higher biomass retention and substrate exploration rates, ultimately resulting in more effective pollutant removal within the Ra‐b system. Moreover, it also demonstrated the critical role of microalgae in promoting environmental stress regulation and system stability. Observations via scanning electron microscopy and optical microscopy revealed the presence of larger aggregates in the Ra‐b system (Figure 3d).

**Figure 4:**
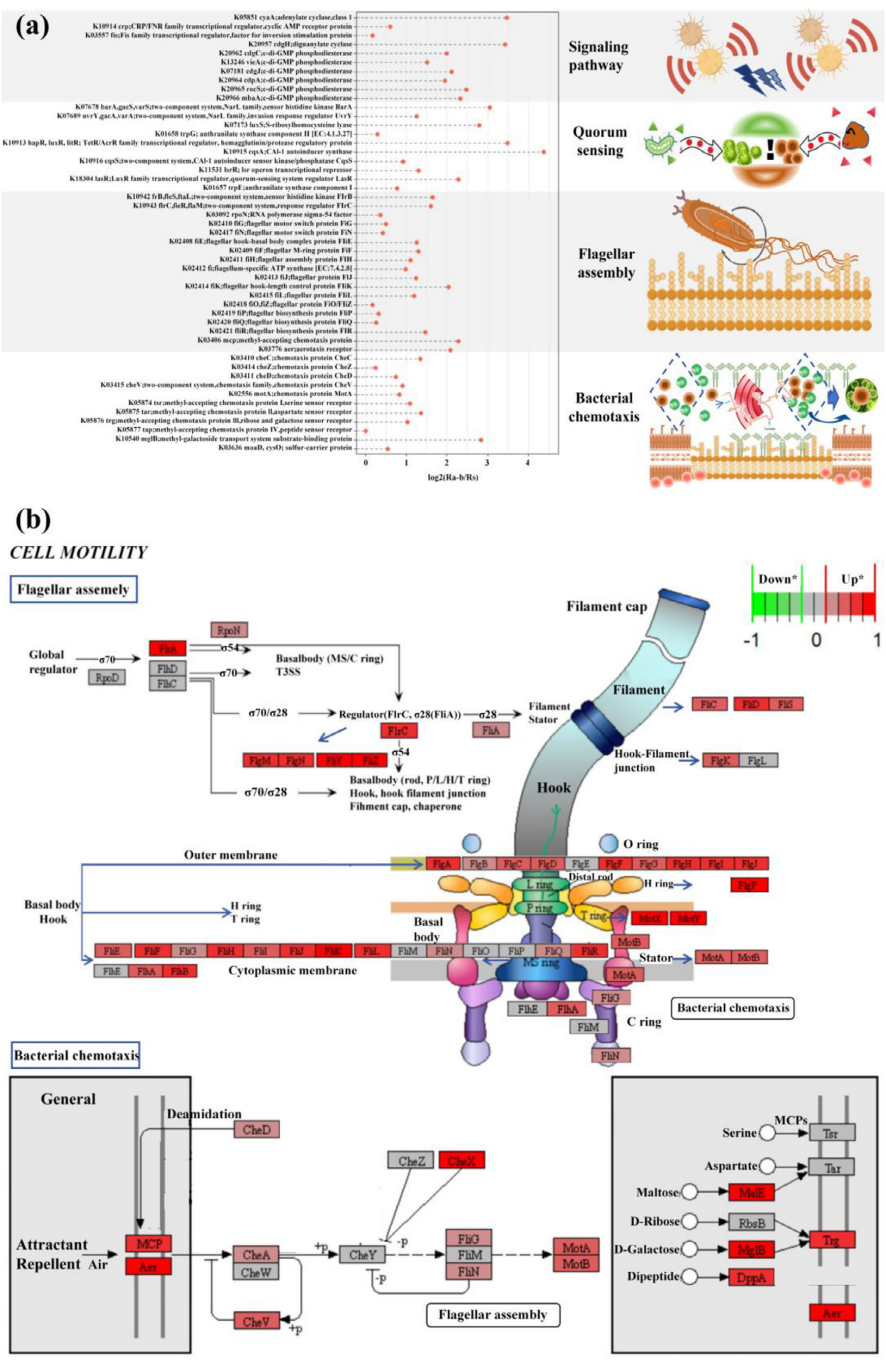
a) Analysis of differential abundance of self‐aggregation associated genes, with the reference ratio pertaining to the comparative genomics between the Ra‐b system and the Rs system. b) Differential analysis of regulation genes related to cellular motility pathways (flagellar movement and chemotaxis) comparing Ra‐b system versus Rs system. Red indicates up‐regulated genes and green indicates down‐regulated genes in the microalgae–bacteria system (padj<0.05).

**Figure 5:**
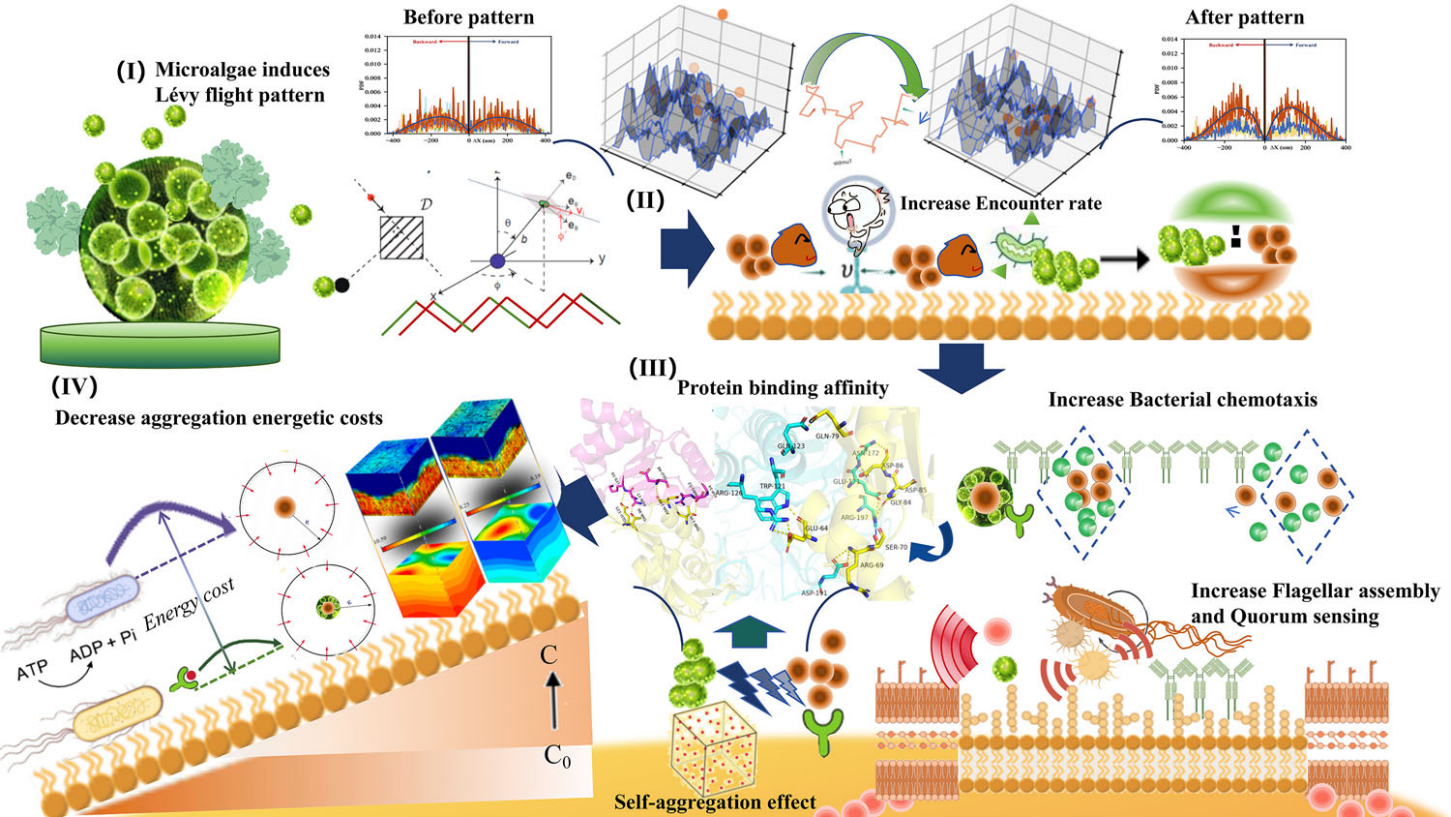
I) Induced by microalgae in the bacteria system, the bacteria motility pattern was altered, enhancing the exploratory distance and random behavior, skewing towards Lévy flight‐type movement. II) This shift augments the bacterial encounter rate and fostering the self‐aggregation of both bacteria‐bacteria and microalgae–bacteria with the concentration of signaling molecules and biomass increasing. III) The enhanced protein binding affinity precipitated the upregulation of pathways related to cellular motility characteristics, such as quorum sensing and flagellar movement, further facilitating self‐aggregation. IV) This process decreased the energetic costs of aggregation and reallocates protein resources to augment other biological functions, providing a crucial foundation and viable strategies for the system's stable operation and efficient pollutant removal.

Our experiments demonstrated that it promoted cellular motility, enhancing self‐aggregation and granulation. Metagenomic analysis revealed that microalgae markedly influenced the abundance of genes related to cellular motility pathways, including bacterial chemotaxis and flagellar assembly^[^ ^25^, ^53^ ^]^ (**Figure** **4**a,b; Figure S8, Supporting Information). The upregulation of these genes is crucial for self‐aggregation and implicated in modulation of collective behavior and environmental response;^[^ ^30^, ^32^, ^54^, ^55^ ^]^ this resulted in a marked enhancement of the abundance of genes in the self‐aggregation pathway (padj<0.01), which is corroborated by reactor operations. Both reactor operations and metagenomic analysis further demonstrate that microalgae enhance intercellular aggregation through altering bacterial cell motility characteristics (padj<0.01) (Figure 4a,b). Specifically, microalgae activate critical genes in the cellular motility pathway, significantly increasing bacterial self‐aggregation effect and the granulation process. Notably, protein docking analysis revealed a significant binding affinity between prominently expressed bacterial proteins (such as SAT and Cbi transcriptional regulators amino acid synthetases) and microalgae (Figure S6, Supporting Information), with binding energies registering as unfavorable (‐384/544 kJ mol^−1^), denoting spontaneous association under natural conditions,^[^ ^56^ ^]^ as a distinctive advantage for self‐aggregation processes (**Figure** **5**).

In addition, metagenomic analysis revealed the regulating pathways of microalgae in enhancing substrate utilization, pollutant removal, and system stability. Metagenomic outcomes indicated a significant increase in the abundance of genes associated with regulatory functions, such as TreC, DsdX, and PTS,^[^ ^57^, ^58^, ^59^ ^]^ as well as critical enzymes for carbon fixation like RbcL and sensor genes on cell membranes^[^ ^60^ ^]^ (padj <0.05) (Figure S7, Supporting Information). The altered motility patterns simulated by the model are reflected in actual reactor operations, confirming that microalgae can enhance bacterial cells' perceptiveness and sensitivity to substrate concentrations. In the microalgae–bacteria system, there was a significant increase in the gene abundance coding for enzymes essential for maintaining system stability (such as GarB, NAD(P)H‐quinone oxidoreductase, ndhE, TCS) and regulatory response enzymes (like EvgS and BvgS)^[^ ^61^, ^62^, ^63^, ^64^ ^]^ (Figure S6, Supporting Information), conducive to sustaining the stability of the biosystem. This stability was also reflected in practical operations; as the operational phase progressed, the bacterial system experienced granule collapses and reduced settling ability, whereas the microalgae–bacteria aggregates displayed larger aggregates and improved settling ability.

Correlation analysis further determined a significant relationship between the self‐aggregation propensity induced by microalgae and the efficiency of pollutant removal and the system stability. This underscores that microalgae, by modulating bacterial motility and enhancing self‐aggregation, not only facilitate the removal of pollutants but are also imperative for ensuring the long‐term stability of the system's operation. In the microalgae–bacteria system, compared to the control bacteria system, there was an increase of 20.0% in biomass, 27.3% in signaling molecule concentrations, 19.5% in self‐aggregation, and 16.7% in nitrogen removal efficiency. These practical results are consistent with results from our bioreactor cell motility model based on ASM3 dynamic processes coupled with cell motility simulation, demonstrating the efficacy of the model. The model can effectively simulate cellular trajectories, biomass accumulation, and removal efficiencies, providing theoretical underpinnings for effective regulatory control of the bioreactor system.

### 2.4 Self‐Aggregation and Symbiosis Regulation Mechanism through Microalgae‐Induced Lévy Flight

In this study, we used the microalgae–bacteria system as a case study to investigate the cell motility characteristics of self‐aggregation mechanisms, which are pivotal for microalgae–bacteria symbiosis and system stability.^[^ ^1^, ^4^, ^5^, ^11^, ^51^ ^]^ We identify microalgae can intensify the Lévy flight behavior in bacteria by altering movement patterns, as evidenced by enhanced randomness and exploratory behavior, increased motility speed, and a heavier‐tailed velocity distribution (Figure 1), thereby intensifying the concentration of signaling molecules and biomass (Figure 2). Consequently, there is a substantial increase in the bacterial encounter rates, culminating in self‐aggregation (Figure 4).

This study then progressed to explore the underlying mechanisms for which microalgae influence bacterial motility (Figure 5). We discovered a significantly robust affinity between specific bacteria and microalgae within the system, characterized by a notable binding energy (‐300 KJ mol^−1^ and 515 KJ mol^−1^) conducive to spontaneous association under naturalistic conditions. Moreover, the presence of microalgae contributes to an increased negative charge within the system, further enhancing an environment conducive to affinity due to the neutralizing actions and the abundance of amino groups within proteins. This affinity alters the configuration of bacterial surface receptors, potentiate genes associated with flagellar locomotion and quorum sensing, and activates signal transduction pathways about cell motility (Figure 4). The propulsive force generated by the flagellar rotation and the consequential alterations in fluid flow modulate near‐field and far‐field flow characteristics, subsequently orchestrating the kinetics of particulates and cellular motility.^[^ ^31^ ^]^

## 3 Discussion

Here, we integrated an effective model of cellular motility in wastewater biological system. This work overcomes limitations imposed by the inherent complexity of wastewater treatment biological systems in studying cellular movement characteristics. Through coordinated parameter interactions and metabolism‐movement coupling, this framework provides new avenues for expanding our understanding of physical phenomena and optimizing the functionality of biological systems. It reveals microscopic mechanisms to optimize energy efficiency and system stability through microecological manipulation, enhancing environmental adaptability and functional performance within biological systems. Specifically, identification and regulation of bacterial motility patterns at the microscale level for beneficial microbial interactions, ultimately enabling macroscopic reactor performance improvements, such as enhanced self‐aggregation effects, for further stable operation and improved pollutant removal. This holds significant application value in the wastewater treatment field, particularly for biological treatment technologies in resource‐limited environments or specialized wastewater treatment, ultimately achieving efficient and cost‐effective microscale regulation.

Our integrated model has great potential for specific pollutant treatment processes. For nitrogen‐rich wastewater, the Lévy flight pattern enhances nitrification and denitrification by enabling nitrifying bacteria to locate and cluster around ammonia‐rich hotspots. In carbon‐limited environments with low C/N ratios, the diffusive search behavior allows efficient exploitation of scarce organic carbon sources. For heavy metal contaminated wastewater, the intermittent, long‐step characteristics of Lévy flights help microbial populations navigate while avoiding prolonged contact with toxic heavy metals. In antibiotic‐laden wastewater, such as hospital or pharmaceutical effluents, this motility pattern enables bacterial populations to maintain heterogeneous spatial distributions, reducing localized overexposure to antibiotics. Moreover, the method can be extended to diverse bioreactor systems, including activated sludge, biofilm reactors, and anaerobic digestion systems. Ultimately, microecological manipulation represents a paradigm shift from macro‐level parameter control to microecological regulation, providing new insights applicable across diverse biological systems and multiple pollutant treatment scenarios.

Our study reveals that microalgae can alter the cellular movement patterns towards Lévy flights, thereby enhancing the self‐aggregation effect, system stability, and pollutant removal with reduced energy consumption. It proved the optimized microalgae–bacteria system ensemble adept at substantially curtailing the energetic expenditure of cellular processes. Based on coarse‐grained models,^[^ ^27^, ^65^ ^]^ compared to a bacterial system at identical parameters, it reduces 10^15^–10^19^ ATP consumption, thereby economizing on the cost of chemotaxis‐related proteins (Note S5, Supporting Information). The resources saved through this optimization can be reallocated to support cell growth or other vital protein functions, enabling bacteria to maintain adequate motility even at low concentrations of chemical attractants.^[^ ^27^ ^]^ This paradigm shift fortifies the overall efficiency of the biological system, thereby ensuring stable operation and effective pollutant removal.

Furthermore, our integrated model for investigating cellular motility in wastewater treatment is significant for enhancing bacterial adaptability to spatially heterogeneous environments and advancing the spatial evolutionary trajectory of microbial consortia, thereby facilitating efficient and economical microscale management of wastewater.^[^ ^66^, ^67^ ^]^ Microbial cells navigate their environment and manage their growth, influencing evolution through movement.^[^ ^68^ ^]^ Establishing a response mechanism between motility patterns and genomic dynamics allows us to institute targeted regulatory mechanisms across a broader range of biological systems. By optimizing complex locomotion patterns, such as Lévy flight, we can manipulate the coexistence of cellular populations at the microscale,^[^ ^21^, ^22^, ^69^ ^]^ regulating population states under specific environmental conditions, such as in the application of specialized wastewater treatment involving antibiotic challenges.^[^ ^67^ ^]^ It introduces innovative strategies for environmental adaptation and functional performance within intricate biological networks, ultimately leading to efficient and cost‐effective microscale management of these systems.

## 4 Experimental Section

### Framework for Dynamic Modeling of Cellular Movement in Bioreactors

We employed the ASM3‐microalgae simulation framework to develop a 3D cellular motility model in a wastewater biosystem. The modeling framework consists of two integrated components based on the Java‐based iDynaMics platform: a metabolic module and a movement module. This extended framework achieves synchronized simulation of microbial growth metabolism and movement behaviors through coordinated parameter interactions and unified time‐step iterations. The model establishes three functional layers: the sludge or sludge‐microalgae layer, an intermediate boundary layer, and the mixed liquid layer providing nutrients (Note S1 and Figure S1, Supporting Information). These layers are integrated via a diffusion boundary interface to facilitate solute transition between the zones.

The model simulates metabolic processes using widely‐validated Monod kinetics, capturing the growth, metabolism, and energy storage of heterotrophs, autotrophs, and microalgae under various conditions (aerobic, anoxic, and endogenous respiration). The framework tracks the dynamics of concentration fields, signal molecule distributions, and biomass concentrations, with all parameters calibrated against experimental reactor operation. Further details on these processes and parameters can be found in Note S1 and Tables S1–S4 (Supporting Information). Specifically, Table S1 (Supporting Information) lists process kinetic rate equations for substrate uptake and biomass growth; Table S2 (Supporting Information) details the matrix of stoichiometric parameters for process rates; Table S3 (Supporting Information) provides values of stoichiometric parameters; Table S4 (Supporting Information) summarizes values of biokinetic, chemical, and physical parameters. Additional methodological details are provided in Note S1 (Supporting Information): Method of Model Construction and Movement Dynamic Simulation.

The metabolism‐movement coupling is achieved through synchronized time‐step iterations with an alternating solution strategy. During each iteration cycle, the metabolic module first updates solute concentration fields and metabolic states based on cell agents' local environment. The movement module then calculates cell positions and particle size changes in response to the updated chemical gradients under physical and biochemical influences, resulting in changes to cellular spatial positions, aggregation effect, and biomass distribution. These spatial redistributions subsequently modify the local metabolic environment, establishing new boundary conditions for the next iteration cycle. This iterative feedback mechanism ensures tight coupling between metabolic processes and movement behaviors, enabling accurate simulation of complex dynamics in bioreactor systems. Detailed model parameters and methods are provided in Note S1 (Supporting Information)Sensitivity Analysis and Model Verification

To ensure model reliability, we conducted sensitivity analysis using the variance‐based Sobol method on 53 independent variables, quantifying model uncertainties into probability distributions. Using Monte Carlo sampling,^[^ ^32^ ^]^ we generated 10800 sample datasets to construct the parameter matrix. Detailed method was stated in Note S2 (Supporting Information). By analyzing the influence of parameters, we identified 8 key parameters with contribution rates exceeding 20% to results (Note S2, Supporting Information).

Model validation was performed through multi‐level verification system indicators, incorporating microscopic indicators (bacterial biomass, microalgal biomass, signal molecule concentrations, and granule sizes), while macroscopic validation assessed removal efficiencies of TN, NH_4_‐N, and COD. Model validation utilized different term experiments (5–20 d) to optimize parameters by comparing model predictions with experimental data using coefficient of determination (*R* ^2^). Then, model robustness was verified through 200 simulations with random initial conditions across varying agent populations (200–2000 units), Each system was evaluated through a multidimensional assessment framework comprising 6–7 indicators in each biosystem, including pollutant removal, biomass concentration, and average particle size, demonstrating high prediction accuracy (*R* ^2^ > 0.97) (Figure S10, Supporting Information). Based on the validated model, we conducted 70‐day simulations and bioreactor operation to investigate movement characteristics of both ABGS and AGS, revealing their impacts on system performance. Through comprehensive integration of model predictions, bioreactor operation results and metagenomic analysis, we explored the intrinsic relationships between microbial movement behaviors, granulation processes and pollutant removal. This multidimensional analysis provided deep insights into the movement‐driven mechanisms of granular systems, enabling comparative analysis of cellular trajectories between bacterial and microalgae–bacteria system.

### Movement Index

The comprehensive assessment of Lévy flight dynamics involves the following indicators: step length autocorrelation, indicating memory effects and dependence structure within the trajectory;^[^ ^44^, ^70^ ^]^ Diffusion exponent used to describe a parameter reflecting the particles diffusion characteristic, particularly in the context of stochastic processes and random walks.^[^ ^45^, ^46^ ^]^ Aggregation Metrics We combined k‐means++ and DBSCAN algorithms for robust cluster analysis. Correlation Dimension was Calculated using Grassberger‐Procaccia algorithm. The correlation dimension is a characteristic measure that describe the geometry of chaotic attractors. It is defined using the correlation sum *C(r)*, the fraction of pairs of points *Xi* in the phase space whose distance is smaller than *r*.^[^ ^47^, ^48^, ^71^ ^]^

The walking properties was further evaluated by analyzing the autocorrelation function or the correlation coefficient of the cell trajectory, indicating memory effects and dependence structure within the trajectory.

(1) $$R_{h}=\frac{C_{h}}{C_{0}}$$

*C*_h_: autocovariance function

(2) $$C_{h}=\frac{1}{N}\;\sum_{t\;=1}^{N-h}\left(Y_{t}-\bar{Y}\right)\left(Y_{t+h}-\bar{Y}\right)$$

C_0_: variance function

(3) $$C_{0}=\frac{\sum_{t=1}^{N}{\left(Y_{t}-\bar{Y}\right)}^{2}}{N}$$

herein, *D* denotes correlation dimension, N delineates the quantity of embedded vectors. The double summation takes into account all unique vector pairs for which “i” is less than “j.”. The Heaviside step function, Θ, assumes a value of one when the argument is positive; otherwise, it is zero. The notation (||X(i) – X(j)||) signifies the Euclidean distance between the vectors “X(i)” and “X(j)”.

The correlation dimension is a characteristic measure that describe the geometry of chaotic attractors. It is defined using the correlation sum *C(r)*, the fraction of pairs of points *Xi* in the phase space whose distance is smaller than r.^[^ ^47^, ^48^, ^71^ ^]^ To describe the spatial coverage and complexity and irregularity of the trajectory and the geometric complexity of the path geometry complexity. The correlation dimension serves as a metric characterizing the intricate geometric attributes of trajectory paths. Specifically:

(4) $$C\left(r\right)\;∼r^{\wedge }D*$$

(5) $$C\;\left(r\right)=\left(2/N\left(N-1\right)\right)*\sum \sum \Theta \left(r-\left|\left|X\left(i\right)\;-\;X\left(j\right)\right|\right|\right)$$

Herein, D denotes Correlation Dimension, N delineates the quantity of embedded vectors. The double summation takes into account all unique vector pairs for which “i” is less than “j”. The Heaviside step function, Θ, assumes a value of one when the argument is positive; otherwise, it is zero. The notation (||X(i) – X(j)||) signifies the Euclidean distance between the vectors “X(i)” and “X(j)”.

Diffusion Exponent can be estimated by analyzing the relationship between the mean squared displacement (MSD) of particles and the corresponding time intervals. The Diffusion Exponent is often considered as the slope of the linear relationship between time intervals and the MSD.

The diffusion exponent: fitted from the MSD calculation:

(6) $$\begin{array}{ccc} \mathrm{MSD}\;\left(\Delta t\right) & = & \frac{1}{N}\;\sum_{i\;=1}^{N}\left({\left(r_{\mathrm{ix}}\left(t+\Delta t\right)-r_{\mathrm{ix}}\left(t\right)\right)}^{2}\right.\\ & & \left.+{\left(r_{\mathrm{iy}}\left(t+\Delta t\right)-r_{\mathrm{iy}}\left(t\right)\right)}^{2}+{\left(r_{\mathrm{iz}}\left(t+\Delta t\right)-r_{\mathrm{iz}}\left(t\right)\right)}^{2}\right) \end{array}$$

Here, N is the Number of agent particles, r_i_ is the 3 D coordinates of the corresponding time.

For aggregation degree and encounter rate analysis, the trajectory of two randomly selected cellular organisms achieving equilibrium is analyzed under the system's boundary conditions. An average of 500 random selections is computed to quantify the average encounter rate. For aggregation degree analysis, clustering tendency and spatial distribution of cells based on incorporated DBSCAN with K‐Means++ resulted in trajectory data features that are more focused and less noisy.^[^ ^72^ ^]^

Trajectory aggregation degree based on clustering tendency and spatial distribution of cells based on K‐means++ to assess collective behavior and compare aggregation degree of two systems. The selection method for K initial cluster centers involves choosing the data point farthest from the current cluster center as the next cluster center, until K initial cluster centers have been selected. Euclidean distance between the spatial coordinate data and the cluster center:

(7) $$d\;\left(X,C_{i}\right)=\sqrt{\sum_{j=1}^{m}{\left(X_{j}-C_{\mathrm{ij}}\right)}^{2}}$$

Here, *X* means Path space coordinate data; *C_i_* is the *i* th cluster center, *X_j_* and *C_ij_* denote corresponding temporal attribute components

Sum of squared errors (SSE) of trajectory for each iteration:

(8) $$\mathrm{SSE}\;=\sum_{i=1}^{k}\sum_{X\in C_{i}}{\left|d\left(X,C_{i}\right)\right|}^{2}$$

Here, k is the number of cluster centers; X denotes the trajectory coordinate dataset.

The Hill estimator plot was used to estimate the tail exponent of a heavy‐tailed distribution.^[^ ^49^, ^73^ ^]^ Specific methods and details refer to the Note S3 (Supporting Information).

For aggregation degree and encounter rate analysis, the trajectory of two randomly selected cellular organisms achieving equilibrium is analyzed under the system's boundary conditions. An average of 500 random selections is computed to quantify the average encounter rate. For aggregation degree analysis, clustering tendency and spatial distribution of cells based on incorporated DBSCAN with K‐Means++ resulted in trajectory data features that are more focused and less noisy.^[^ ^72^ ^]^

Lévy flight efficiency was quantified by comparing the net displacement of cells to the signal gradient they experience, based on the difference between the concentration signal molecules between the new motion position and the original position of the cell agent in each single time step tending to the steady state.

(9) $$\mathrm{Levy}\;\mathrm{efficiency}=\frac{\sum_{t_{i}}^{t_{n}}\sum_{n_{0}}^{n_{r}}\left(D_{e}-D_{s}\right)}{\sum_{t_{i}}^{t_{n}}\sum_{n_{0}}^{n_{r}}\left(C_{e}-C_{s}\right)}$$

Here, C_e_‐C_s_denotes normalized signal molecule concentration difference; C_s_ denotes signal molecule concentrations at the original positions; C_e_ denotes signal molecule concentrations at the new positions. D_e_‐D_s_represents Euclidean distances from the signal source to the new and original positions.

### Reactor Operation, Aggregation Potential and Signaling Molecules

We operated two sequencing batch reactors (SBRs): a granular sludge reactor (*R*s) and a microalgae–bacteria granular sludge reactor (Ra‐b), each with a 1.5 L volume, through an oxic‐anoxic‐oxic‐settling cycle for 70 d. The cycle included phases of aeration, no aeration, and settling, varying from 40 to 55 min, with light exposure for the Ra‐b system at a 12 h day/night regime. Each SBR completed six 4 h cycles daily. Specific details refer to Note S4 (Supporting Information). Water quality, mixed liquor suspended solids (MLSS), mixed liquor volatile suspended solids (MLVSS), settling velocity (SV) and SVI were measured according to the Standard Method (APHA).^[^ ^74^ ^]^ Sludge morphologies were imaged using a stereo microscope SZX7 mounted with a cooled digital color camera DP72 (Olympus Co., Japan) for phenotypic analysis and analyzed using the software Image‐Pro Plus 6.0 (Media Cybernetics Inc, USA). Characterization of N‐acyl homoserine lactones (AHL) as signaling molecules as autoinducers of QS regulation was measured from established methods.^[^ ^75^, ^76^ ^]^ Granule size distribution was determined by a laser particle analyzer (QICPIC, Sympatec, Germany). The method of Wang et al.^[^ ^77^ ^]^ was used to determine the sludge aggregation capacity. Supernatants from two systems were taken at operation times every weekDNA Extraction, Metagenomics Sequencing and Protein Docking Analysis

DNA samples were extracted from Rs and Ra‐b using the magnetic bead method and purified through lysis, digestion, and adsorption. A metagenomic library was constructed and sequenced on the Illumina HiSeq platform. Sequence read quality was optimized via Trimmomatic, and de novo assembly was performed with Spades.

Functional metagenomic analysis utilized the KEGG database and KofamKOALA tool, leveraging HMMER for enhanced accuracy over BLAST. Gene abundance was quantified through alignment with BWA‐MEM and subsequent coverage analysis. Community composition was determined using DIAMOND to compare protein sequences against the NCBI NR database, with taxonomic lineage traced through NCBI's Taxonomy database.^[^ ^78^ ^]^ The NR annotation rate was 86%, with an effective species annotation rate over 82%.

Protein docking was performed between microalgae and significantly differentially expressed proteins to investigate the relationships using GRAMM‐X. The protein structural domains were obtained from the Protein Data Bank PDB database. Pymol (Version 2.4) and PDBePISA were used to investigate protein–protein interactions and further visual analysis.^[^ ^56^ ^]^

### Statistical Analysis

Differential gene abundance analysis first normalizes the raw read counts to eliminate technical bias between samples and then calculates the log_2_ fold change (FC) of the normalized data to quantify relative changes in gene abundance across different samples. The comparisons between Rs and Ra‐b were carried out using one‐way variance analysis (ANOVA) followed by Tukey's multiple comparisons tests or Kruskal‐Wallis test and Dunn's multiple comparisons tests to determine the differences. To control for multiple hypothesis testing, we applied the Benjamini‐Hochberg method to correct *P* values and false discovery rates (*q*‐values) for calculations.^[^ ^79^ ^]^ All statistical significance tests in the experiments were two‐sided, and results were considered statistically significant when the *P* value and FDR *q* value were < 0.05. Error bars were defined as SD (*n* = 3, replicates), detailed description refers to Note S4 (Supporting Information).

## Conflict of Interest

The authors declare no conflict of interest.

## Supporting information



Supporting Information

## Data Availability

The data that support the findings of this study are available from the corresponding author upon reasonable request.
