# Supplementary material for: Movement Mechanisms Harness Lévy Flight for Energy‐Efficient Wastewater Treatment in Microalgae–Bacteria Systems
Source: Adv Sci (Weinh). 2025 Sep 8;12(44):e04676. doi: 10.1002/advs.202504676 (PMC12667456; doi:10.1002/advs.202504676)
Supplement: Supplementary file 1 — Supporting Information [file ADVS-12-e04676-s001.docx]

**Supplementary Material**

**SUPPLEMENTARY NOTE 1: Method of Model Construction and Movement Dynamic Simulation**

**Model Framework, Boundary Conditions, and Coupled Computational Implementation**

As shown in SUPPLEMENTARY Figure 1, the ASM3-Microalgae framework model establishes three functional layers: the granular sludge layer with microalgae serving as the primary carrier of active biomass and core region for biological reactions; the boundary layer mediating mass transfer and gradient formation between wastewater and cells; and the bulk liquid layer providing substrates and nutrients for the system. The model employs cyclic boundary conditions, ensuring that when agent units exceed specified boundaries, they re-enter the system from opposing boundaries—a design that maintains mass continuity while accurately simulating the mixing dynamics of cylindrical reactors.

The framework integrates microalgae-bacterial growth dynamics with cell movement modeling through unified time-step coupling. During initialization, the model establishes states for solutes, particles, reactions, species, and solvers based on operational parameters. The system monitors agent unit lifecycle states: generation, division (triggered when cell radius reaches division threshold), and removal (when external forces reduce radius below survival threshold). Within each time step, the model employs an alternating solution strategy: the metabolic module first updates solute concentration fields and metabolic states, followed by the movement module calculating cell positions and morphological changes based on the updated environment, ensuring synchronized updates of metabolic states and movement behaviors.

In the dynamic coupling process, the kinetic module updates solute concentration fields and metabolic states, generating concentration gradients that drive agent unit movements and alter their grid positions, with biomass concentration gradients influencing the direction and velocity of microalgae-bacterial agents. Spatial distribution changes of agent units, through growth, decay, and division processes, affect local biomass concentrations, which further feedback to concentration gradient dynamics. Based on this, movement simulation performs dynamic updates and constraints, implementing initialization, movement restrictions, and position-shape updates. The dynamic distribution of solute concentrations serves both as motility drivers and growth constraints, simulating changes in cell positions and granule morphology, while movement-induced spatial distribution changes reciprocally affect solute diffusion and metabolism. The model alternately solves solute and agent grids, comprehensively considering biological dynamics, physical and chemical interactions, dynamically simulating complex ecological processes in algae-bacterial granular systems.

Within the bioreactor framework, parameters output from the biological reaction model serve as input parameters for the movement simulation, while movement states and biological distributions act as conditions for biological reactions. In each integration step, the final position of cells is determined by the combined effects of biological dynamics, physical and chemical interactions (including fluid dynamics) ^65^. This innovative coupled computational framework achieves dynamic synergy between metabolic processes and movement behaviors, enabling accurate simulation of complex ecological processes in microalgae-bacterial granular systems.

**Model Components and Process Analysis**

1. Biological Component Network

Based on the ASM3-Microalgae framework, we constructed a comprehensive biological component network encompassing autotrophic bacteria, heterotrophic bacteria responsible for organic matter degradation, and photoautotrophic microalgae (XA). These biological components participate in system material cycling and energy conversion through growth, respiration, and decay processes. Metabolic products include endogenous storage materials (XSTo), inert organic matter (XI), and extracellular polymeric substances (EPS). EPS, continuously produced during microbial metabolism, undergoes hydrolysis to form soluble organic matter (SBAP, SUAP) that can be utilized by heterotrophic bacteria. The metabolic kinetics and stoichiometric parameters for all components are detailed in Table 1-4.

1. Dynamic Metabolic Network Simulation

In the autotrophic bacterial network, XAOB utilizes NH_4_^+^-N as electron donor and O_2_ as electron acceptor under aerobic conditions, oxidizing NH_4_^+^-N to NO_2_^-^ while synthesizing new biomass (Table 1-1). During endogenous respiration, XAOB oxidizes intracellular storage compounds using O_2_, producing CO_2_ and NH_4_^+^ (Table 1-2). Under anoxic conditions, XAOB can utilize NO_2_^-^ and NO_3_^-^ as electron acceptors, reducing NO_2_^-^ to N_2_ and NO_3_- to NO_2_- respectively (Table 1-3,1-4). During decay, cellular components are converted to inert organic matter XI and NH_4_^+^-N through endogenous respiration (Table 1-27). XNOB primarily oxidizes NO_2_- to NO_3_- under aerobic conditions (Table 1-5), with similar endogenous respiration and decay processes (Table 1-6,1-7,1-8,1-28).

The heterotrophic bacterial (X_H_) metabolic network involves three major processes: storage, growth, and respiration. During the storage phase, X_H_ converts NH_4_+-N and soluble organic matter (SS) to endogenous storage material X_STo_ under aerobic conditions (Table 1-9), or accomplishes denitrification while converting S_S_ to X_STo_ under anaerobic conditions using NO_2_^-^ and NO_3_^-^ as electron acceptors (Table 1-10,1-11). During growth, X_H_ can either utilize S_S_ directly for aerobic growth (Table 1-12) or reduce NO_2_^-^ to N2 and NO_3_^-^ to NO_2_^-^ through denitrification under anoxic conditions (Table 1-14,1-15). Additionally, X_H_ can grow aerobically and anoxically using stored X_STo_ (Table 1-13,1-16,1-17). The respiration phase includes both X_STo_ aerobic and anaerobic respiration (Table 1-18,1-19,1-29) and X_H_'s endogenous respiration and decay (Table 1-20,1-21,1-22,1-26).

Microalgae (X_A_) primarily removes nitrogen, phosphorus, and other nutrients through photosynthesis, absorption, and diffusion processes, while releasing O_2_ for other microorganisms during respiration (Table 1-23-25). The EPS produced during metabolism can be hydrolyzed to soluble organic matter (SBAP, SUAP) and further utilized by heterotrophic bacteria.

1. Dynamic Microbial Movement Simulation

We employed a comprehensive movement simulation framework based on fluid-phase microbial models, including physical forces (hydrodynamic forces, Equations 1-3; diffusion effects, Equation 4) and biochemical effects. The biochemical driving process comprises three mechanisms: initial exploration using a tetrahedral framework (Equations 5-6) integrating random movement with concentration gradient effects; chemotactic adjustment introducing movement bias compensation (Equations 7-8) for trajectory optimization; and final decision stage synthesizing local signal molecule and substrate concentration gradients (Equation 9) to ensure movement rationality. The microbial dynamics process encompasses physical effects of fluid dynamics and diffusion, along with biochemical influences.

Physical Force and Interactions

Fluid Dynamics: In the fluid dynamics of the biosystems, for enhanced clarity and brevity, the hydrodynamic thrust, R, applied to a cellular agent is conceptualized through the subsequent relationships:

$\text{R=0.5}\text{C}_{\text{d}}\text{A}_{\text{Cr}}\text{ρ}\text{v}^{2}$ (1)

Here, $C_{d}$ denotes Drag coefficient pertinent to the cellular agent;$A_{\mathrm{Cr}}$ represents cross-sectional area of the agent;$\rho$is the fluid density; v is the velocity of fluid. Consequently, the acceleration a of the cellular agent ascribed to the influence of fluid dynamics is articulated as:

$a=\frac{R}{m_{t}}$ (2)

Here, R denotes hydrodynamic force, and m is the mass of the cellular agent at this time. Thus, the displacement engendered by the fluid dynamic impact upon the motility of the cellular entity is articulated as:

$\text{dis=}\text{v}_{0}\text{Δt}\text{+}\frac{1}{2}\text{a}\text{Δt}^{2}$ (3)

Diffusion Effects: Moreover, our model has incorporated various contributing factors to the diffusion process, encompassing diffusion enhancement due to near-field locomotion as well as physical diffusion effects catalyzed by fluid motion, like those arising from far-field flow effects, and both of which influentially modulate the state of cellular motility^32^.

$\text{D}_{\text{eff}}\text{=ε}\text{(D}_{0}\text{+}\text{α}_{\text{WJ}}\text{N}_{\text{c}}\text{+}\frac{\text{L}^{2}}{2}\text{γ}\text{ν}_{\text{i}}\text{N}_{\text{c}}\text{)}$ (4)

Here, D_0_ is standard diffusion coefficient; $\alpha_{\mathrm{WJ}}$ represents contribution of the far-field effects to the diffusion; Nc is microbes’ concentrations; L is the average of bacteria jump; γ denotes the cross-sectional area of microorganisms；v_i_ is the speed of different types of organisms at the previous moment. This coupling enables dynamic adjustment of diffusion intensity in response to changes in biomass, cellular growth states, and energy metabolism levels.

The biochemical driving process comprises three mechanisms: initial exploration integrating random motion with chemical cues, chemotactic adjustment incorporating roll bias compensation, and final movement correction responding to local gradients.

In the initial exploration stage, tetrahedral framework integrates four key factors: random movement components ensuring spatial exploration, substrate concentration gradients providing chemical cues, signal molecule distributions mediating cell-cell communication, and biomass density effects reflecting local environment constraints. Here, cell behavior is construed as a tetrahedral framework, reflecting the confluence of randomness, substrate concentration, and signalling molecule concentration on cell movement. The agent cells adjust their motility strategies in response to the local substrate, emulating preliminary exploratory behaviors driven by biochemical cues:

$CoeCon=3\frac{AC}{h}$ (5)

Step=distEacRun·(1+αCoeCon)·U(0,1) (6)

Here, α denotes response degree of substrate concentration; CoeCon is concentration convergence; A is area of the triangle; C is half circumference of the triangle; h represents vertical height from vertex to bottom triangle.

In the chemotactic adjustment phase, cells recalibrate their movement trajectories and displacements through a roll bias compensation mechanism. Following exploratory behavior, cells recalibrate their movement trajectory and displacement in alignment with the concentration gradient of chemotactic agents on their surface, thus executing a biased random walk. This mechanism optimizes movement trajectories through three key parameters: spatial competition compensation factor (β), adjustment duration (Δt), and average movement velocity (VT). The system exhibits characteristic run-and-tumble behavior patterns, with periodic forward movement interrupted by reorientation events. These movement patterns maintain dynamic association with cellular metabolic states, ensuring that motility strategies can promptly respond to environmental changes. Schnitzer differentiated between "non-adaptive pseudotaxis" and "true adaptive chemotaxis" using a kinetic approach, analyzing the steady-state behavior across various scenarios^18^. Our model integrates both the transient and steady-state behaviors of chemotaxis as influenced by exploratory actions, characterized by a periodic succession of forward runs punctuated by abrupt tumbling reorientations^12^. For decoding gradients of concentration spatially, which in turn shapes their navigational tactics, the chemotactic response function is as follows:

$\text{Rall Bias=}\text{β}\Delta t\bar{v_{t}}\frac{{{c\left( x,y,z \right)}_{t}}^{+}-{{c\left( x,y,z \right)}_{t}}^{-}}{{{c\left( x,y,z \right)}_{t}}^{+}+{{c\left( x,y,z \right)}_{t}}^{-}}$ (7)

Here,$\beta$represents spatial competitive motion offset compensation;$\Delta t$ is the bias duration; $\bar{v_{t}}$ denotes the average velocity; ${{c\left( x,y,z \right)}_{t}}^{+}$ means the total number of positive moves toward the matrix gradient at time t${{; c\left( x,y,z \right)}_{t}}^{-}$is the total number of times moving in the negative direction toward the matrix gradient at time t.

In the final decision-making phase, movement is determined through a comprehensive gradient-based mechanism. The model synthesizes signal molecule gradients (∇c) and substrate concentration gradients (∇s), optimizing movement direction vectors through adjustment coefficients (α,β). Each cell evaluates concentration gradients at its specific location and determines its trajectory based on inherent chemotactic preferences (positive or negative). The movement distance is then modulated by ambient substrate levels and density thresholds.

$\vec{\text{D}}\text{=∂}\text{∇}\text{s+β}\text{∇}\text{c}$ (8)

Here, $\vec{D}$is the direction vector of cell movement; α, β represent positive adjust coefficient; ∇c is the direction in greatest signal molecule concentration gradient and ∇s is the direction in greatest substrate concentration gradient.

Table.1 Process kinetic rate equations

| **Number** | **Process** | **Kinetics rates expressions** |
| --- | --- | --- |
| 1 | Aerobic growth of $X_{AOB}$ | $u_{max}\frac{S_{{{NH}_{4}}^{+}}}{S_{{{NH}_{4}}^{+}}+K_{{NH}_{4},AOB}}\frac{S_{O_{2}}}{S_{O_{2}}+K_{O_{2,}AOB}}X_{AOB}$ |
| 2 | Aerobic endogenous respiration of $X_{AOB}$ | $b_{AOB{,O}_{2}}\frac{S_{O_{2}}}{S_{O_{2}}+K_{O_{2},AOB}}X_{AOB}$ |
| 3 | Anoxic endogenous respiration of $X_{AOB}$ on ${{NO}_{2}}^{-}$ | $y_{Nend}b_{AOB}\frac{K_{O_{2},AOB}}{S_{O_{2}}+K_{O_{2},AOB}}\frac{S_{{{NO}_{2}}^{-}}}{K_{{NO}_{2},AOB+}S_{{{NO}_{2}}^{-}}}X_{AOB}$ |
| 4 | Anoxic endogenous respiration of $X_{AOB}$ on ${{NO}_{3}}^{-}$ | $y_{Nend}b_{AOB}\frac{K_{O_{2},AOB}}{S_{O_{2}+}K_{O_{2},AOB}}\frac{S_{{{NO}_{3}}^{-}}}{K_{{NO}_{3},AOB+}S_{{{NO}_{3}}^{-}}}X_{AOB}$ |
| 5 | Aerobic growth of $X_{NOB}$ | $u_{NOB}\frac{S_{{{NO}_{2}}^{-}}}{S_{{{NO}_{2}}^{-}}+K_{{NO}_{2}NOB}}\frac{S_{O_{2}}}{S_{O_{2}}+K_{O_{2},NOB}}\frac{S_{{{NH}_{4}}^{+}}}{S_{{{NH}_{4}}^{+}}+K_{{NO}_{2}NOB}}X_{NOB}$ |
| 6 | Aerobic endogenous respiration of $X_{NOB}$ | $b_{NOB,O_{2}}\frac{S_{O_{2}}}{S_{O_{2}}+K_{O_{2},NOB}}X_{NOB}$ |
| 7 | Anoxic endogenous respiration of $X_{NOB}$ on ${{NO}_{2}}^{-}$ | $y_{Nend}b_{NOB}\frac{K_{O_{2},NOB}}{S_{O_{2}}+K_{O_{2},NOB}}\frac{S_{{{NO}_{2}}^{-}}}{S_{{{NO}_{2}}^{-}}+K_{{NO}_{2}NOB}}X_{NOB}$ |
| 8 | Anoxic endogenous respiration of $X_{NOB}$ on ${{NO}_{3}}^{-}$ | $y_{Nend}b_{NOB}\frac{K_{O_{2},NOB}}{S_{O_{2}}+K_{O_{2},NOB}}\frac{S_{{{NO}_{3}}^{-}}}{S_{{{NO}_{3}}^{-}}+K_{{NO}_{3}NOB}}X_{NOB}$ |
| 9 | Aerobic storage of $X_{H}$ | $k_{STO}\frac{S_{O_{2}}}{K_{O_{2},H}+S_{O_{2}}}\frac{S_{s}}{K_{S,H}+S_{s}}X_{H}$ |
| 10 | Anoxic storage of $X_{H}$ on $S_{s}$(${{NO}_{2}}^{-}$-$N_{2}$) | $y_{H,{NO}_{2}}k_{STO}\frac{K_{O_{2},H}}{K_{O_{2},H}+S_{O_{2}}}\frac{S_{{{NO}_{2}}^{-}}}{K_{{NO}_{2},H}+S_{{{NO}_{2}}^{-}}}\frac{S_{s}}{K_{S,H}+S_{s}}X_{H}$ |
| 11 | Anoxic storage of $X_{H}$ on $S_{s}({{NO}_{3}}^{-}$-${{NO}_{2}}^{-}$) | $y_{H,{NO}_{3}}k_{STO}\frac{K_{O_{2},H}}{K_{O_{2},H}+S_{O_{2}}}\frac{S_{{{NO}_{3}}^{-}}}{K_{{NO}_{3}H}+S_{{{NO}_{3}}^{-}}}\frac{S_{s}}{K_{S,H}+S_{s}}X_{H}$ |
| 12 | Aerobic growth of $X_{H}$ on $S_{s}$ | $u_{H,S_{s}}\frac{S_{O_{2}}}{K_{O_{2},H}+S_{O_{2}}}\frac{S_{{{NH}_{4}}^{+}}}{S_{{{NH}_{4}}^{+}}+K_{{NH}_{4}H}}\frac{S_{s}}{K_{S,H}+S_{s}}X_{H}$ |
| 13 | Aerobic growth of $X_{H}$ on $X_{STO}$ | $u_{H,STO}\frac{S_{O_{2}}}{K_{O_{2}H}+S_{O_{2}}}\frac{S_{{{NH}_{4}}^{+}}}{S_{{{NH}_{4}}^{+}}+K_{{NH}_{4}H}}\frac{K_{S,H}}{K_{S,H}{+S}_{s}}\frac{X_{STO}/X_{H}}{(K_{STO,H}+X_{STO})/X_{H}}X_{H}$ |
| 14 | Anoxic growth of $X_{H}$ on $S_{s}$(${{NO}_{2}}^{-}$-$N_{2}$) | $y_{H,{NO}_{2}}u_{H,S_{s}}\frac{K_{O_{2},H}}{K_{O_{2},H}+S_{O_{2}}}\frac{S_{{{NH}_{4}}^{+}}}{S_{{{NH}_{4}}^{+}}+K_{{NH}_{4},H}}\frac{S_{{{NO}_{2}}^{-}}}{K_{{NO}_{2},H}+S_{{{NO}_{2}}^{-}}}\frac{S_{s}}{K_{S,H}+S_{s}}X_{H}$ |
| 15 | Anoxic growth of $X_{H}$ on $S_{s}({{NO}_{3}}^{-}$-${{NO}_{2}}^{-}$) | $y_{H,{NO}_{3}}u_{H,S_{s}}\frac{K_{O_{2},H}}{K_{O_{2},H}+S_{O_{2}}}\frac{S_{{{NH}_{4}}^{+}}}{S_{{{NH}_{4}}^{+}}+K_{{NH}_{4},H}}\frac{S_{{{NO}_{3}}^{-}}}{K_{{NO}_{3},H}+S_{{{NO}_{3}}^{-}}}\frac{S_{s}}{K_{S,H}+S_{s}}X_{H}$ |
| 16 | Anoxic growth of $X_{H}$ on $X_{STO}({{NO}_{3}}^{-}$-${{NO}_{2}}^{-}$) | $y_{H,{NO}_{3}}u_{H,STO}\frac{K_{O_{2},H}}{K_{O_{2},H}+S_{O_{2}}}\frac{S_{{{NH}_{4}}^{+}}}{S_{{{NH}_{4}}^{+}}+K_{{NH}_{4},H}}\frac{S_{{{NO}_{3}}^{-}}}{K_{{NO}_{3},H}+S_{{{NO}_{3}}^{-}}}\frac{K_{S,H}}{K_{S,H}+S_{s}}\frac{X_{STO}/X_{H}}{(K_{STO,H}+X_{STO})/X_{H}}X_{H}$ |
| 17 | Anoxic growth of $X_{H}$ on $X_{STO}$(${{NO}_{2}}^{-}$-$N_{2}$) | $y_{H,{NO}_{2}}u_{H,STO}\frac{K_{O_{2},H}}{K_{O_{2},H}+S_{O_{2}}}\frac{S_{{{NH}_{4}}^{+}}}{S_{{{NH}_{4}}^{+}}+K_{{NH}_{4},H}}\frac{S_{{{NO}_{2}}^{-}}}{K_{{NO}_{2},H}+S_{{{NO}_{2}}^{-}}}\frac{K_{S,H}}{K_{S,H}+S_{s}}\frac{X_{STO}/X_{H}}{(K_{STO,H}+X_{STO})/X_{H}}X_{H}$ |
| 18 | Anoxic respiration of $X_{STO}$ on nitrite | $y_{Hend,NO2}b_{STO,{NO}_{2}}\frac{K_{O_{2},H}}{K_{O_{2},H}+S_{O_{2}}}\frac{S_{{{NO}_{2}}^{-}}}{K_{{NO}_{2},H}+S_{{{NO}_{2}}^{-}}}X_{STO}$ |
| 19 | Anoxic respiration of $X_{STO}$ on nitrate | $y_{Hend,NO3}b_{STO,{NO}_{3}}\frac{K_{O_{2},H}}{K_{O_{2},H}+S_{O_{2}}}\frac{S_{{{NO}_{3}}^{-}}}{K_{{NO}_{3},H}+S_{{{NO}_{3}}^{-}}}X_{STO}$ |
| 20 | Aerobic endogenous respiration of $X_{H}$ | $b_{H,O_{2}}\frac{S_{O_{2}}}{K_{O_{2},H}+S_{O_{2}}}X_{H}$ |
| 21 | Anoxic respiration of $X_{H}$(${{NO}_{2}}^{-}$-$N_{2}$) | $y_{Hend,NO2}b_{{H,NO}_{2}}\frac{K_{O_{2},H}}{K_{O_{2},H}+S_{O_{2}}}\frac{S_{{{NO}_{2}}^{-}}}{K_{{NO}_{2},H}+S_{{{NO}_{2}}^{-}}}X_{H}$ |
| 22 | Anoxic respiration of $X_{H}({{NO}_{3}}^{-}$-${{NO}_{2}}^{-}$) | $y_{Hend,NO3}b_{{H,NO}_{3}}\frac{K_{O_{2},H}}{K_{O_{2},H}+S_{O_{2}}}\frac{S_{{{NO}_{3}}^{-}}}{K_{{NO}_{3},H}+S_{{{NO}_{3}}^{-}}}X_{H}$ |
| 23 | Growth of $X_{A}$ | $u_{A}\frac{S_{{{NH}_{4}}^{+}}}{S_{{{NH}_{4}}^{+}}+K_{{NH}_{4}A}}X_{A}$ |
| 24 | Aerobic respiration of $X_{A}$ | $b_{A,O_{2}}\frac{S_{O_{2}}}{K_{O_{2},A}+S_{O_{2}}}X_{A}$ |
| 25 | Decay of $X_{A}$ | $b_{A}X_{A}$ |
| 26 | Decay of $X_{H}$ | $b_{H}X_{H}$ |
| 27 | Decay of $X_{AOB}$ | $b_{AOB}X_{AOB}$ |
| 28 | Decay of $X_{NOB}$ | $b_{NOB}X_{NOB}$ |
| 29 | Aerobic respiration of $X_{STO}$ | $b_{STO,O_{2}}\frac{S_{O_{2}}}{K_{O_{2},H}+S_{O_{2}}}X_{STO}$ |

Table.2 Matrix of stoichiometric parameters for process rates

| **Process rates** | $\mathbf{S}_{{\mathbf{NH}_{\mathbf{4}}}^{\mathbf{+}}}$ | $\mathbf{S}_{{\mathbf{NO}_{\mathbf{2}}}^{\mathbf{-}}}$ | $\mathbf{S}_{{\mathbf{NO}_{\mathbf{3}}}^{\mathbf{-}}}$ | $\mathbf{S}_{\mathbf{O}_{\mathbf{2}}}$ | $\mathbf{S}_{\mathbf{s}}$ | $\mathbf{S}_{\mathbf{N}_{\mathbf{2}}}$ | $\mathbf{X}_{\mathbf{AOB}}$ | $\mathbf{X}_{\mathbf{NOB}}$ | $\mathbf{X}_{\mathbf{H}}$ | $\mathbf{X}_{\mathbf{STO}}$ | $\mathbf{X}_{\mathbf{A}}$ |
| --- | --- | --- | --- | --- | --- | --- | --- | --- | --- | --- | --- |
| Aerobic growth of $X_{\mathrm{AOB}}$ | P1,1 | P1,2 |  | P1,4 |  |  | P1,7 |  |  |  |  |
| Aerobic endogenous respiration of $X_{\mathrm{AOB}}$ | P2,1 |  |  | P2,4 |  |  | P2,7 |  |  |  |  |
| Anoxic endogenous respiration of $X_{\mathrm{AOB}}$ on ${\mathrm{NO}_{2}}^{-}$ | P3,1 | P3,2 |  |  |  | P3,6 | P3,7 |  |  |  |  |
| Anoxic endogenous respiration of $X_{\mathrm{AOB}}$ on ${\mathrm{NO}_{3}}^{-}$ | P4,1 |  | P4,3 |  |  | P4,6 | P4,7 |  |  |  |  |
| Aerobic growth of $X_{\mathrm{NOB}}$ | P5,1 | P5,2 | P5,3 | P5,4 |  |  |  | P5,8 |  |  |  |
| Aerobic endogenous respiration of $X_{\mathrm{NOB}}$ | P6,1 |  |  | P6,4 |  |  |  | P6,8 |  |  |  |
| Anoxic endogenous respiration of $X_{\mathrm{NOB}}$ on ${\mathrm{NO}_{2}}^{-}$ | P7,1 | P7,2 |  |  |  | P7,6 |  | P7,8 |  |  |  |
| Anoxic endogenous respiration of $X_{\mathrm{NOB}}$ on ${\mathrm{NO}_{3}}^{-}$ | P8,1 |  | P8,3 |  |  | P8,6 |  | P8,8 |  |  |  |
| Aerobic storage of $X_{H}$ | P9,1 |  |  | P9,4 | P9,5 |  |  |  |  | P9,10 |  |
| Anoxic storage of $X_{H}$ on $S_{s}$(${\mathrm{NO}_{2}}^{-}$-$N_{2}$) | P10,1 | P10,2 |  |  | P10,5 | P10,6 |  |  |  | P10,10 |  |
| Anoxic storage of $X_{H}$ on $S_{s}({\mathrm{NO}_{3}}^{-}$-${\mathrm{NO}_{2}}^{-}$) | P11,1 | P11,2 | P11,3 |  | P11,5 |  |  |  |  | P11,10 |  |
| Aerobic growth of $X_{H}$ on $S_{s}$ | P12,1 |  |  | P12,4 | P12,5 |  |  |  | P12,9 |  |  |
| Aerobic growth of $X_{H}$ on $X_{\mathrm{STO}}$ | P13,1 |  |  | P13,4 |  |  |  |  | P13,9 | P13,10 |  |
| Anoxic growth of $X_{H}$ on $S_{s}$(${\mathrm{NO}_{2}}^{-}$-$N_{2}$) | P14,1 | P14,2 |  |  | P14,5 | P14,6 |  |  | P14,9 |  |  |
| Anoxic growth of $X_{H}$ on $S_{s}({\mathrm{NO}_{3}}^{-}$-${\mathrm{NO}_{2}}^{-}$) | P15,1 | P15,2 | P15,3 |  | P15,5 |  |  |  | P15,9 |  |  |
| Anoxic growth of $X_{H}$ on $X_{\mathrm{STO}}({\mathrm{NO}_{3}}^{-}$-${\mathrm{NO}_{2}}^{-}$) | P16,1 | P16,2 | P16,3 |  |  |  |  |  | P16,9 | P16,10 |  |
| Anoxic growth of $X_{H}$ on $X_{\mathrm{STO}}$(${\mathrm{NO}_{2}}^{-}$-$N_{2}$) | P17,1 | P17,2 |  |  |  | P17,6 |  |  |  | P17,10 |  |
| Anoxic respiration of $X_{\mathrm{STO}}$ on nitrite |  | P18,2 |  |  |  | P18,6 |  |  |  | P18,10 |  |
| Anoxic respiration of $X_{\mathrm{STO}}$ on nitrate |  | P19,2 | P19,3 |  |  |  |  |  |  | P19,10 |  |
| Aerobic endogenous respiration of $X_{H}$ | P20,1 |  |  | P20,4 |  |  |  |  | P20,9 |  |  |
| Anoxic respiration of $X_{H}$(${\mathrm{NO}_{2}}^{-}$-$N_{2}$) | P21,1 | P21,2 |  |  |  | P21,6 |  |  | P21,9 |  |  |
| Anoxic respiration of $X_{H}({\mathrm{NO}_{3}}^{-}$-${\mathrm{NO}_{2}}^{-}$) | P22,1 | P22,2 | P22,3 |  |  |  |  |  | P22,9 |  |  |
| Growth of $X_{A}$ | P23,1 |  |  | P23,4 |  |  |  |  |  |  | P23,11 |
| Aerobic respiration of $X_{A}$ | P24,1 |  |  | P24,4 |  |  |  |  |  |  | P24,11 |
| Decay of $X_{A}$ | P25,1 |  |  |  |  |  |  |  |  |  | P25,11 |
| Decay of $X_{H}$ |  |  |  |  |  |  |  |  | P26,9 |  |  |
| Decay of $X_{\mathrm{AOB}}$ |  |  |  |  |  |  | P27,7 |  |  |  |  |
| Decay of $X_{\mathrm{NOB}}$ |  |  |  |  |  |  |  | P28,8 |  |  |  |
| Aerobic respiration of $X_{\mathrm{STO}}$ |  |  |  |  |  |  |  |  |  | P29,10 |  |

Table.3 Values of stoichiometric parameters（S11-S17）

| **Stoichiometric coefficients** | |
| --- | --- |
| P1,1 | $-1$ |
| P1,2 | $1$ |
| P1,4 | $-(3.43-Y_{\mathrm{AOB}})$ |
| P1,7 | $Y_{\mathrm{AOB}}$ |
| P2,1 | $i_{N,BM}-f_{X_{I}}i_{N,XI}$ |
| P2,4 | $-(1-f_{X_{I}})$ |
| P2,7 | $-1$ |
| P3,1 | $i_{N,BM}-f_{X_{I}}i_{N,XI}$ |
| P3,2 | $-(1-f_{X_{I}})/1.71$ |
| P3,6 | $(1-f_{X_{I}})/1.71$ |
| P3,7 | $-1$ |
| P4,1 | $i_{N,BM}-f_{X_{I}}i_{N,XI}$ |
| P4,3 | $-(1-f_{X_{I}})/2.86$ |
| P4,6 | $(1-f_{X_{I}})/2.86$ |
| P4,7 | $-1$ |
| P5,1 | $-i_{N,BM}$ |
| P5,2 | $-1$ |
| P5,3 | $1$ |
| P5,4 | $-(1.41-Y_{\mathrm{NOB}})$ |
| P5,8 | $Y_{\mathrm{NOB}}$ |
| P6,1 | $i_{N,BM}-f_{X_{I}}i_{N,XI}$ |
| P6,4 | $-(1-f_{X_{I}})$ |
| P6,8 | $-1$ |
| P7,1 | $i_{N,BM}-f_{X_{I}}i_{N,XI}$ |
| P7,2 | $-(1-f_{X_{I}})/1.71$ |
| P7,6 | $(1-f_{X_{I}})/1.71$ |
| P7,8 | $-1$ |
| P8,1 | $i_{N,BM}-f_{X_{I}}i_{N,XI}$ |
| P8,3 | $-(1-f_{X_{I}})/2.86$ |
| P8,6 | $(1-f_{X_{I}})/2.86$ |
| P8,8 | $-1$ |
| P9,1 | ${-i}_{N,Ss}$ |
| P9,4 | $-(1-Y_{STO,O_{2}})$ |
| P9,5 | $-1$ |
| P9,10 | $Y_{STO,O_{2}}$ |
| P10,1 | ${-i}_{N,Ss}$ |
| P10,2 | $-(1-Y_{STO,\mathrm{NO}_{2}})/1.71$ |
| P10,5 | $-1$ |
| P10,6 | $(1-Y_{STO,\mathrm{NO}_{2}})/1.71$ |
| P10,10 | $Y_{STO,\mathrm{NO}_{2}}$ |
| P11,1 | ${-i}_{N,Ss}$ |
| P11,2 | $(1-Y_{STO,\mathrm{NO}_{3}})/2.86$ |
| P11,3 | $-(1-Y_{STO,\mathrm{NO}_{3}})/2.86$ |
| P11,5 | $-1$ |
| P11,10 | $Y_{STO,\mathrm{NO}_{3}}$ |
| P12,1 | ${-i}_{N,BM}$ |
| P12,4 | $1-1/Y_{H,O_{2}}$ |
| P12,5 | $-1/Y_{H,O_{2}}$ |
| P12,9 | $1$ |
| P13,1 | ${-i}_{N,Ss}$ |
| P13,4 | $-(1-Y_{H,STO})$ |
| P13,9 | $1$ |
| P13,10 | $-1/Y_{H,STO}$ |
| P14,1 | $-i_{N,BM}$ |
| P14,2 | $-(1-Y_{H,NO_{2}})/(1.72Y_{H,NO_{2}})$ |
| P14,5 | $-1/Y_{H,O_{2}}$ |
| P14,6 | $(1-Y_{H,NO_{2}})/(1.72Y_{H,NO_{2}})$ |
| P14,9 | $1$ |
| P15,1 | ${-i}_{N,BM}$ |
| P15,2 | $(1-Y_{H,NO_{3}})/(1.14Y_{H,NO_{3}})$ |
| P15,3 | $-(1-Y_{H,NO_{3}})/(1.14Y_{H,NO_{3}})$ |
| P15,5 | $-1/Y_{H,O_{2}}$ |
| P15,9 | $1$ |
| P16,1 | ${-i}_{N,BM}$ |
| P16,2 | $(1-Y_{H,STO})/(1.14Y_{H,STO})$ |
| P16,3 | $-(1-Y_{H,STO})/(1.14Y_{H,STO})$ |
| P16,9 | $1$ |
| P16,10 | $-1/Y_{H,STO}$ |
| P17,1 | ${-i}_{N,BM}$ |
| P17,2 | $-(1-Y_{H,STO})/(1.72Y_{H,STO})$ |
| P17,6 | $(1-Y_{H,STO})/(1.72Y_{H,STO})$ |
| P17,10 | $-1/Y_{H,STO}$ |
| P18,2 | $-1/1.71$ |
| P18,6 | $1/1.71$ |
| P18,10 | $-1$ |
| P19,2 | $1/2.86$ |
| P19,3 | $-1/2.86$ |
| P19,10 | $-1$ |
| P20,1 | $i_{N,BM}-f_{X_{I}}i_{N,XI}$ |
| P20,4 | $-(1-f_{X_{I}})$ |
| P20,9 | $-1$ |
| P21,1 | $i_{N,BM}-f_{X_{I}}i_{N,XI}$ |
| P21,2 | $-(1-f_{X_{I}})/1.71$ |
| P21,6 | $(1-f_{X_{I}})/1.71$ |
| P21,9 | $-1$ |
| P22,1 | $i_{N,BM}-f_{X_{I}}i_{N,XI}$ |
| P22,2 | $(1-f_{X_{I}})/2.86$ |
| P22,3 | $-(1-f_{X_{I}})/2.86$ |
| P22,9 | $-1$ |
| P23,1 | ${-i}_{N,A}$ |
| P23,4 | $8/3i_{C,A}+8i_{H,A}-i_{O,A}-12/7(i_{N,A})$ |
| P23,11 | $1$ |
| P24,1 | $i_{N,A}-f_{A}i_{N,A}$ |
| P24,4 | ${(i}_{O,A}-f_{A}i_{O,A})-8(i_{H,A}-f_{A}i_{H,A})-8/3(i_{C,A}-f_{A}i_{C,A})+12/7(i_{N,A}-f_{A}i_{N,A})$ |
| P24,11 | $-1$ |
| P25,1 | $i_{N,A}-\left( 1-f_{A} \right)Y_{A}i_{N,XS}-f_{A}Y_{A}i_{N,A}$ |
| P25,11 | $-1$ |
| P26,9 | $-1$ |
| P27,7 | $-1$ |
| P28,8 | $-1$ |
| P29,10 | $-1$ |

Table.4 Values of biokinetic, chemical and physical parameters

| **Parameters** | **Description** | **Value** | **Unit** | **Source(h)/Calibrated** |
| --- | --- | --- | --- | --- |
| $u_{\max}$ | Maximum specific growth rate of $X_{\mathrm{AOB}}$ | 0.12 | $h^{-1}$ | Calibrated |
| $K_{\mathrm{NH}_{4},AOB}$ | Saturation constant of $X_{\mathrm{AOB}}$ on $S_{{NH_{4}}^{+}}$ | 0.5 | $\mathrm{gNm}^{-3}$ | 0.5 (S1) |
| $K_{O_{2,}\mathrm{AOB}}$ | Saturation constant of $X_{\mathrm{AOB}}$ on $S_{O_{2}}$ | 0.5 | ${go_{2}m}^{-3}$ | 0.5 (S1) |
| $b_{\mathrm{AOB}{,O}_{2}}$ | Endogenous respiration rate of $X_{\mathrm{AOB}}$ | 0.01 | $h^{-1}$ | 0.01 (S2) |
| $y_{\mathrm{Nend}}$ | coefficient of correction | 0.1 | $-$ | Calibrated |
| $K_{\mathrm{NO}_{2},AOB}$ | Saturation constant of $X_{\mathrm{AOB}}$ on $S_{{NO_{2}}^{-}}$ | 0.5 | $\mathrm{gNm}^{-3}$ | 0.5 (S3) |
| $K_{\mathrm{NO}_{3},AOB}$ | Saturation constant of $X_{\mathrm{AOB}}$ on $S_{{NO_{3}}^{-}}$ | 0.5 | $\mathrm{gNm}^{-3}$ | 0.5 (S3) |
| $u_{\mathrm{NOB}}$ | Maximum specific growth rate of $X_{\mathrm{NOB}}$ | 0.108 | $h^{-1}$ | Calibrated |
| $K_{\mathrm{NO}_{2}\mathrm{NOB}}$ | Saturation constant of $X_{\mathrm{NOB}}$ on $S_{{NO_{2}}^{-}}$ | 5.5 | $\mathrm{gNm}^{-3}$ | 5.5 (S4) |
| $K_{O_{2},NOB}$ | Saturation constant of $X_{\mathrm{NOB}}$ on $S_{O_{2}}$ | 0.47 | ${go_{2}m}^{-3}$ | 0.47 (S5) |
| $b_{NOB,O_{2}}$ | Endogenous respiration rate of $X_{\mathrm{NOB}}$ | 0.004 | $h^{-1}$ | Calibrated |
| $K_{\mathrm{NO}_{3},NOB}$ | Saturation constant of $X_{\mathrm{NOB}}$ on $S_{{NO_{3}}^{-}}$ | 0.5 | $\mathrm{gNm}^{-3}$ | 0.5 (S3) |
| $k_{\mathrm{STO}}$ | Storage rate constant | 0.523 | $h^{-1}$ | 0.523 (S6) |
| $K_{O_{2},H}$ | Saturation constant of $X_{H}$ on $S_{O_{2}}$ | 0.2 | ${go_{2}m}^{-3}$ | 0.2 (S7) |
| $K_{S,H}$ | Saturation constant of $X_{H}$ on $S_{s}$ | 20 | $\mathrm{gCODm}^{-3}$ | 20 (S7) |
| $y_{H,\mathrm{NO}_{2}}$ | coefficient of correction | 0.15 | $-$ | Calibrated |
| $K_{\mathrm{NO}_{2},H}$ | Saturation constant of $X_{H}$ on $S_{NO_{2}}$ | 0.5 | $\mathrm{gNm}^{-3}$ | 0.5 (S7) |
| $y_{H,\mathrm{NO}_{3}}$ | coefficient of correction | 0.15 | $-$ | Calibrated |
| $K_{\mathrm{NO}_{3}H}$ | Saturation constant of $X_{H}$ on $S_{NO_{3}}$ | 0.5 | $\mathrm{gNm}^{-3}$ | 0.5 (S7) |
| $u_{H,S_{s}}$ | Maximum specific growth rate for $S_{s}$ of $X_{H}$ | 0.07 | $h^{-1}$ | 0.07 (S6) |
| $K_{\mathrm{NH}_{4}H}$ | Saturation constant of $X_{H}$ on $S_{NH_{4}}$ | 0.01 | $\mathrm{gNm}^{-3}$ | 0.01 (S7) |
| $u_{H,STO}$ | Maximum specific growth rate for $X_{\mathrm{STO}}$ of $X_{H}$ | 0.06 | $h^{-1}$ | Calibrated |
| $K_{STO,H}$ | Saturation constant of $X_{H}$ on $X_{\mathrm{STO}}$ | 1.0 | $\mathrm{gCODm}^{-3}$ | 1.0 (S7) |
| $y_{Hend,NO2}$ | coefficient of correction | 0.35 | $-$ | Calibrated |
| $b_{STO,\mathrm{NO}_{2}}$ | Anoxic respiration rate for $S_{{NO_{2}}^{-}}$ of $X_{\mathrm{STO}}$ | 0.004 | $h^{-1}$ | 0.004 (S7) |
| $y_{Hend,NO_{3}}$ | coefficient of correction | 0.25 | $-$ | Calibrated |
| $b_{STO,\mathrm{NO}_{3}}$ | Anoxic respiration rate for $S_{{NO_{3}}^{-}}$ of $X_{\mathrm{STO}}$ | 0.004 | $h^{-1}$ | 0.004 (S7) |
| $b_{STO,O_{2}}$ | Aerobic respiration rate for $X_{\mathrm{STO}}$ | 0.008 | $h^{-1}$ | 0.008 (S7) |
| $b_{{H,NO}_{2}}$ | Anoxic respiration rate coefficient for $S_{{NO_{2}}^{-}}$ of $X_{H}$ | 0.004 | $h^{-1}$ | 0.004 (S7) |
| $b_{{H,NO}_{3}}$ | Anoxic respiration rate coefficient for $S_{{NO_{3}}^{-}}$ of $X_{H}$ | 0.004 | $h^{-1}$ | 0.004 (S7) |
| $u_{A}$ | Maximum specific growth rate of $X_{A}$ | 0.08 | $h^{-1}$ | Calibrated |
| $K_{\mathrm{NH}_{4},A}$ | Saturation constant of $X_{A}$ on $S_{NH_{4}}$ | 1.0 | $\mathrm{gNm}^{-3}$ | Calibrated |
| $b_{H,O_{2}}$ | Endogenous respiration rate of $X_{H}$ | 0.004 | $h^{-1}$ | 0.004 (S8,S10) |
| $K_{O_{2},A}$ | Saturation constant of $X_{A}$ respiration on $S_{O_{2}}$ | 0.2 | $\mathrm{gOm}^{-3}$ | 0.2 (S1) |
| $b_{A}$ | Decay rate of $X_{A}$ | 0.001 | $h^{-1}$ | Calibrated |
| $b_{H}$ | Decay constant of $X_{H}$ | 0.004 | $h^{-1}$ | 0.004 (S10) |
| $b_{\mathrm{AOB}}$ | Decay constant of $X_{\mathrm{AOB}}$ | 0.01 | $h^{-1}$ | 0.01 (S2) |
| $b_{\mathrm{NOB}}$ | Decay constant of $X_{\mathrm{NOB}}$ | 0.003 | $h^{-1}$ | Calibrated |
| $Y_{\mathrm{AOB}}$ | Yield of $X_{\mathrm{AOB}}$ | 0.13 | $\mathrm{gCODgCOD}^{-1}$ | 0.13 (S1) |
| $i_{C,BM}$ | Fraction of carbon in biomass | 0.323 | $\mathrm{gCgCO}D^{-1}$ | 0.323 (S1) |
| $i_{N,BM}$ | Fraction of nitrogen in biomass | 0.075 | $\mathrm{gNgCOD}^{-1}$ | 0.075 (S1) |
| $i_{N,XI}$ | Fraction of nitrogen in $X_{I}$ | 0.016 | $\mathrm{gNgCOD}^{-1}$ | 0.016 (S1) |
| $i_{C,BM}$ | Fraction of carbon in biomass | 0.323 | $\mathrm{gCgCOD}^{-1}$ | 0.323 (S1) |
| $f_{\mathrm{XI}}$ | Production of $X_{I}$ in endogenous respiration of biomass | 0.2 | $\mathrm{gCODgCOD}^{-1}$ | 0.20 (S7) |
| $i_{C,XI}$ | Fraction of carbon in $X_{I}$ | 0.327 | $\mathrm{gCgCOD}^{-1}$ | 0.327 (S1) |
| $Y_{\mathrm{NOB}}$ | Yield of $X_{\mathrm{NOB}}$ | 0.15 | $\mathrm{gCODgCOD}^{-1}$ | Calibrated |
| $i_{N,Ss}$ | Fraction of nitrogen in $S_{S}$ | 0.03 | $\mathrm{gNgCOD}^{-1}$ | 0.03 (S7) |
| $Y_{STO,O_{2}}$ | Aerobic yield coefficient for $X_{\mathrm{STO}}$ of $X_{H}$ | 0.85 | $\mathrm{gCODgCOD}^{-1}$ | 0.85 (S7) |
| $Y_{STO,\mathrm{NO}_{2}}$ | Anoxic yield coefficient for $X_{\mathrm{STO}}$ and $S_{N{O_{2}}^{-}}$ of $X_{H}$ | 0.8 | $\mathrm{gCODgCOD}^{-1}$ | 0.80 (S7) |
| $Y_{STO,\mathrm{NO}_{3}}$ | Anoxic yield coefficient for $X_{\mathrm{STO}}$ and $S_{N{O_{3}}^{-}}$ of $X_{H}$ | 0.8 | $\mathrm{gCODgCOD}^{-1}$ | 0.80 (S7) |
| $Y_{H,O_{2}}$ | Yield of $X_{H}$ | 0.63 | $\mathrm{gCODgCOD}^{-1}$ | 0.63 (S7) |
| $i_{C,S_{s}}$ | Fraction of carbon in SS | 0.318 | $\mathrm{gCgCOD}^{-1}$ | 0.318 (S1) |
| $Y_{H,STO}$ | Yield coefficient for $X_{\mathrm{STO}}$ of $X_{H}$ | 0.85 | $\mathrm{gCODgCOD}^{-1}$ | 0.85 (S6) |
| $Y_{H,NO_{2}}$ | Yield of $X_{H}$ using nitrite as electron acceptor | 0.54 | $\mathrm{gCODgCOD}^{-1}$ | 0.54 (S7) |
| $Y_{H,NO_{3}}$ | Yield of $X_{H}$ using nitrate as electron acceptor | 0.5 | $\mathrm{gCODgCOD}^{-1}$ | 0.5 (S1) |
| $i_{N,A}$ | Fraction of nitrogen in algae | 0.065 | $\mathrm{gNgCOD}^{-1}$ | 0.065 (S9) |
| $i_{C,A}$ | Fraction of carbon in algae | 0.387 | $\mathrm{gCgCOD}^{-1}$ | 0.387 (S1) |
| $i_{H,A}$ | Fraction of hydrogen in algae | 0.075 | $\mathrm{gHgCOD}^{-1}$ | 0.075 (S1) |
| $i_{O,A}$ | Fraction of oxygen in algae | 0.538 | $\mathrm{gOgCOD}^{-1}$ | 0.538 (S1) |
| $f_{A}$ | Production of XI in endogenous respiration of algae | 0.2 | $\mathrm{gCODgCOD}^{-1}$ | 0.2 (S1) |
| $Y_{A}$ | Yield of $X_{A}$ | 0.62 | $\mathrm{gCODgCOD}^{-1}$ | 0.62 (S1) |
| $i_{N,XS}$ | Fraction of nitrogen in $X_{S}$ | 0.034 | $\mathrm{gNgCOD}^{-1}$ | 0.034 (S1) |
| $i_{C,XS}$ | Fraction of carbon in $X_{S}$ | 0.318 | $\mathrm{gCgCOD}^{-1}$ | 0.318 (S1) |
| $b_{A,O_{2}}$ | Endogenous respiration rate of $X_{H}$ | 0.004 | $h^{-1}$ | 0.1 (S1) |

**SUPPLEMENTARY NOTE 2: Model Verification and Sensitivity Analysis of Model Parameters Using the Sobol Method**

Throughout the course of this study, sensitivity analysis was conducted on the model parameters utilizing the Sobol method (S26-28). a total of 10800 sampling samples were employed, each involving 53 independent variables. Using Monte Carlo sampling, we generated 10800 sample datasets to construct the parameter matrix. The Monte Carlo sampling method was employed to generate the sampling matrix, yielding the following dataset. The selection of 10,800 samples was based on the Saltelli sampling scheme, where the total sample size *Ntotal* is calculated as:

(9)

Here, D=53 (number of input parameters in our study), and N is the base sample size per group. According to Saltelli et al, the minimum recommended base sample size N is:

(10)

This would yield a minimum total sample size of:

(11)

However, due to the complex nonlinear interactions among parameters in our system, we increased the base sample size t N=100 to reduce Monte Carlo sampling errors to approximately 10% (estimated via preliminary tests). This adjustment ensures reasonable confidence intervals while balancing computational costs. The final sample size was thus:

(12)

To compute parameter sensitivity, we saved the data for each solute and biomass in the system at various iteration numbers, with each iteration number corresponding to a specific simulation runtime. The Sobol parameter sensitivity analysis method was then applied to calculate the sensitivity of these parameters. Employing the Monte Carlo sampling method (S29, S30), we obtained a substantial volume of random sampling data within the permissible parameter range. This data was subsequently fed into the model for calculation and analysis, ultimately resulting in the derivation of parameter sensitivity within the model.

The first-order sensitivity index quantifies the impact of individual parameters on the outcome, while the second-order sensitivity index captures the interactive effects between pairs of input parameters on the same outcome. Assuming 'x' (x1, x2, …, xi) represents the parameters under analysis, where each parameter, once scaled, resides within the interval [0,1] and is uniformly distributed, and assuming a function 'f(x)' denotes the sensitivity relative to these parameters with a mean of f(0) and a variance 'D', then the first-order contribution of the ith parameter is expressed as:$S_{i}=\frac{D_{i}}{D}$; the second-order contribution between the ith and jth parameters is determined as $S_{\mathrm{ij}}=\frac{D_{\mathrm{ij}}}{D}$

We initially defined specific parameter ranges to serve as sampling intervals. To refine the appropriateness of these predefined sampling intervals, we incorporated upper and lower adjustment intervals based on existing reference data, thereby optimizing the value space. Following the completion of sensitivity sampling across all sampling groups, we computed the average values to gauge the responsiveness of each parameter to the solute. Parameters exhibiting higher sensitivity were singled out for further optimization. These refined parameters were reintroduced into the model for calculation and experimentation. The results were subsequently verified and compared against actual experimental data, allowing for continuous error reduction and refinement of the model's simulation effectiveness.

$\text{Sampling matrix m=}\left[ \begin{matrix} \text{n}_{\text{1,1}} & \text{⋯}\text{n}_{\text{1,i}}\text{⋯}\text{n}_{\text{1,i+1}}\text{⋯} & \text{n}_{\text{1,53}} \\ \text{⋮} & & \text{⋮} \\ \text{n}_{\text{j,1}} & \text{⋯} & \text{n}_{\text{j,533}} \end{matrix} \right]$ (13)

Here, $i$ denotes Argument index number; $j$: Sampling sample number index number; *n* denotes individual sample.

The first-order response index formula:

$\text{S}_{\text{i}}\text{=}\frac{\text{Var}_{\text{Xi}}\text{(}\text{E}_{\text{X\textasciitilde i}}\text{(Y|}\text{X}_{\text{i}}\text{))}}{\text{Var(Y)}}$ (14)

Here, $S_{i}$ denotes the first-order sensitivity index of the i-th parameter, measuring the proportional contribution of the input variable Xi to the output variance, $X_{i}$ denotes the i-th input variable, Y denotes the model output, $E_{X\sim i}(Y|X_{i})$ denotes the conditional expectation of the model output Y with respect to Xi.

Second-Order Sensitivity Indices

$\text{S}_{\text{ij}}\text{=}\frac{\text{Var}_{\text{X}_{\text{i}}\text{X}_{\text{j}}}\left( \text{E}_{\text{X\textasciitilde ij}}\left( \text{Y} | \text{X}_{\text{i}}\text{,}\text{X}_{\text{j}} \right) \right)\text{-}\text{Var}_{\text{X}_{\text{i}}}\left( \text{E}_{\text{X\textasciitilde i}}\left( \text{Y} | \text{X}_{\text{i}} \right) \right)\text{-}\text{Var}_{\text{X}_{\text{j}}}\text{(}\text{E}_{\text{X\textasciitilde j}}\text{(Y|}\text{X}_{\text{j}}\text{))}}{\text{Var(Y)}}$ (15)

Here，${Var}_{X_{i}X_{j}}\left( E_{X\sim ij}\left( Y | X_{i},X_{j} \right) \right)$ denotes the variance of the conditional expectation of the output Y with respect to the variables $X_{i}$ and $X_{j}$，${Var}_{X_{i}}\left( E_{X\sim i}\left( Y | X_{i} \right) \right)$ represents the variance of the conditional expectation of Y with respect to $X_{i}$，$X_{j}$ represents the variance of the conditional expectation of Y with respect to $X_{j}$。$S_{ij}$ denotes the proportion of the output variance caused by the interaction between input variables $X_{i}$ and $X_{j}$ relative to the total variance.

The total response index formula：

$\text{1=}\sum_{\text{i=1}}^{\text{k}} \text{S}_{\text{i}}\text{+}\sum_{\text{i<j}} \text{S}_{\text{ij}}\text{+}\sum_{\text{i<j<l}} \text{S}_{\text{ij}}\text{+}\text{S}_{\text{1,2,...,k}}$ (16)

Here, each sub-term quantifies the contribution of interactions between Xi and Xj to the total output variance. K denotes the total number of input variables; $\mathcal{S}_{i}$ represents the first-order sensitivity index of Y with respect to $X_{i}$. $\mathcal{S}_{\mathrm{ij}}$ represents the second-order sensitivity index of Y with respect to $X_{i}$ and $X_{j}$, and so on for higher-order terms.

In practical computations, Monte Carlo simulation is employed for estimation:

$\text{Var}_{\text{Xi}}\left( \text{E}_{\text{X\textasciitilde i}}\left( \text{Y} | \text{X}_{\text{i}} \right) \right)\text{≈}\frac{1}{\text{N}}\sum_{\text{j=1}}^{\text{N}} \text{f(B)}_{\text{j}}\text{*}{\text{f(}\text{A}_{\text{B}}^{\text{i}}\text{)}}_{\text{j}}\text{-}\text{f}_{0}^{2}$ (17)

$\text{E}_{\text{X\textasciitilde i}}\left( \text{Y} | \text{X}_{\text{i}} \right)\text{≈}\frac{1}{\text{2N}}\sum_{\text{j=1}}^{\text{N}} {\text{(}{\text{f}\left( \text{A} \right)}_{\text{j}}\text{-}{\text{f(}\text{A}_{\text{B}}^{\text{i}}\text{)}}_{\text{j}}\text{)}}^{2}$ (18)

Here, *N* denotes the number of Monte Carlo sampling points; i and j represent the i-th and j-th sample indices, respectively; ${f(B)}_{j}$ denotes the model output value computed using the j-th sample point from matrix B; ${f(A_{B}^{i})}_{j}$ denotes the model output value computed using the j-th sample point from the hybrid matrix $A_{B}^{i}$; $f_{0}$ represents the estimated mean of the model output; $E_{X\sim i}\left( Y | X_{i} \right)$ denotes the conditional expectation of Y with respect to $X_{i}$; $\mathrm{Var}_{\mathrm{Xi}}\left( E_{X\sim i}\left( Y | X_{i} \right) \right)$ denotes the variance of the conditional expectation of Y with respect to $X_{i}$.

**SUPPLEMENTARY NOTE 3: Cell Movement Trajectory Dynamic Simulation and Evaluation Method**

The comprehensive assessment of Levy flight dynamics involves the following indicators:

Step Length Autocorrelation (S18, 20): With random and irregular and long-term memory effects of Levy flight, the direction of cell movement in its current location may be influenced by its past path. The autocorrelation function evaluates the walking nature (Randomness) and long-term memory (Long-term Memory). The walking properties was further evaluated by analyzing the autocorrelation function or the correlation coefficient of the cell trajectory. Assessing the correlation between consecutive step lengths to understand the persistence or anti-persistence of the random walk, indicating memory effects and dependence structure within the trajectory.

$\text{R}_{\text{h}}\text{=}\frac{\text{C}_{\text{h}}}{\text{C}_{0}}$ (19)

$C_{h}$: autocovariance function

$\text{C}_{\text{h}}\text{=}\frac{1}{\text{N}}\sum_{\text{t=1}}^{\text{N-h}} \text{(}\text{Y}_{\text{t}}\text{-}\bar{\text{Y}}\text{)(}\text{Y}_{\text{t+h}}\text{-}\bar{\text{Y}}\text{)}$ (20)

$C_{0}$: variance function

$\text{C}_{0}\text{=}\frac{\sum_{\text{t=1}}^{\text{N}} {\text{(}\text{Y}_{\text{t}}\text{-}\bar{\text{Y}}\text{)}}^{2}}{\text{N}}$ (21)

Trajectory Aggregation Degree (S25): clustering tendency and spatial distribution of cells based on K-means++ to assess collective behavior and compare aggregation degree of two systems. The selection method for K initial cluster centers involves choosing the data point farthest from the current cluster center as the next cluster center, until K initial cluster centers have been selected. Euclidean distance between the spatial coordinate data and the cluster center:

$\text{d}\left( \text{X,}\text{C}_{\text{i}} \right)\text{=}\sqrt{\sum_{\text{j=1}}^{\text{m}} {\text{(}\text{X}_{\text{j}}\text{-}\text{C}_{\text{ij}}\text{)}}^{2}}$ (22)

X: Path space coordinate data, $C_{i}$: The i th cluster center, $X_{j}$、$C_{\mathrm{ij}}$: Corresponding temporal attribute component

Sum of squared errors (SSE) of trajectory for each iteration：

$\text{SSE=}\sum_{\text{i=1}}^{\text{k}} \sum_{\text{X}\text{∈}\text{C}_{\text{i}}} {\text{|d}\left( \text{X,}\text{C}_{\text{i}} \right)\text{|}}^{2}$ (23)

k: Number of cluster centers, X: trajectory coordinate dataset

Correlation Dimension (S21-23)：To describe the spatial coverage and complexity and irregularity of the trajectory and the geometric complexity of the (Levy flight) path Geometry Complexity. The correlation dimension serves as a metric characterizing the intricate geometric attributes of trajectory paths. By computing the fraction of point pairs within a phase space that are separated by a distance less than a radius 'r', one may derive the correlation sum, C(r), which conforms to a relationship indicative of the system's inherent dimensional complexity. Specifically:

(24)

$\text{C}\left( \text{r} \right)\text{=(2/N(N-1))*}\sum\sum\text{Θ(r - ||X(i) - X(j)||)}$ (25)

Herein, D denotes Correlation Dimension, N delineates the quantity of embedded vectors. The double summation takes into account all unique vector pairs for which 'i' is less than 'j'. The Heaviside step function, Θ, assumes a value of one when the argument is positive; otherwise, it is zero. The notation ( ||X(i) - X(j)|| ) signifies the Euclidean distance between the vectors 'X(i)' and 'X(j)'.

By computing the correlation sum for a gamut of distance thresholds 'r', and performing a linear fit to the logarithmic plot of 'r' against 'C(r)', one quantifies the system's correlation dimension—a quantifier of the trajectory paths' complexity.

Diffusion Exponent (S19, S20) used to describe a parameter that reflects the diffusion characteristics of particles, particularly in the context of stochastic processes and random walks (31). It quantifies the relationship between the particle's displacement over time intervals. In general, the Diffusion Exponent can be estimated by analyzing the relationship between the mean squared displacement (MSD) of particles and the corresponding time intervals. The Diffusion Exponent is often considered as the slope of the linear relationship between time intervals and the MSD.

The diffusion exponent: fitted from the mean square displacement ( MSD) calculation：

$\text{MSD}\left( \text{∆t} \right)\text{=}\frac{1}{\text{N}}\sum_{\text{i=1}}^{\text{N}} \text{(}{\text{(r}_{\text{ix}}\left( \text{t+∆t} \right)\text{-}\text{r}_{\text{ix}}\left( \text{t} \right)\text{)}}^{2}\text{+}{\text{(r}_{\text{iy}}\left( \text{t+∆t} \right)\text{-}\text{r}_{\text{iy}}\left( \text{t} \right)\text{)}}^{2}\text{+}{\text{(r}_{\text{iz}}\left( \text{t+∆t} \right)\text{-}\text{r}_{\text{iz}}\left( \text{t} \right)\text{)}}^{2}\text{)}$ (26)

N: Number of agent particles, $r_{i}$: 3 D coordinates of the corresponding time.

Expected velocity of moving cell:

By analyzing an extensive dataset comprising motion trajectories of individual agents, we derived displacement data at each step's equilibrium state to show probability distribution functions (PDF) of cell displacements. The directional information is determined by calculating the positive and negative differences between the preceding and succeeding positions. Subsequently, the expected displacement is computed through integration over the value interval, and the resultant value is divided by the movement time to yield the expected velocity of moving cell.

$\text{E}\left( \text{V} \right)\text{=}\frac{\int_{\text{min}}^{\text{max}} \text{xPDFdx}}{\text{△}\text{t}}$ (27)

X: Cell movement displacement with equilibrium state; $\triangle t$: Move time with equilibrium state

**SUPPLEMENTARY NOTE 4: Real Reactor Operation**

Two sequence bath reactors (SBR) with working volume of 1.5 L (s of individcm) with oxic-anoxic-oxic-settling (O-A-O-S) operation. A whole operating cycle was 240 min, including 5-min feeding, 40-min aeration, 55-min no aeration, 120-min aeration, 15-min settling, and 5-min decanting (day 1 to day 30), 5 minutes of settling time, and 5 minutes of decanting and 10 minutes of anaerobic time in last 20min (day31 to day70). Seeding sludge was collected from the Wangtang Municipal Wastewater Treatment Plant, Harbin, China, which was acclimated in the SBR before being inoculated into Rs (no algae inoculation) and Ra-b (with algae inoculation) with an initial sludge concentration of 5000±100 mg/L. Ra-b system was inoculated with an algal biomass concentration of approximately 600±10 mg/L. The outer wall of Ra-b system was covered with LED cold white light strips measuring 3m in length, providing a light intensity of approximately 150 μmol/(m2·s) (3000 Lux) from 9:00 a.m. to 9:00 p.m. each day, with a light-dark ratio of 1:1 over a constant illumination period of 12 hours. The temperature of the experimental environment was maintained at approximately 20-25℃, and the pH of the control system was set to 7.5. The aeration volume of the system was maintained at a constant rate of 1 L/min. Each single cycle involved a volume exchange rate of 50%, with a hydraulic retention time of 8 hours. The two reaction systems operated synchronously, with 6 cycles performed each day, and each cycle lasting for 4 hours.

Table. 6 Components of reactor synthetic sewage

| Component | Concentration (mg/L) | Component | Concentration (mg/L) |
| --- | --- | --- | --- |
| Glucose | 50 | H_3_BO3 | 2.86 |
| CH_3_COONa | 150 | MnCl2 ·4H_2_O | 1.86 |
| NaHCO_3_ | 300 | ZnSO4 ·7H_2_O | 0.22 |
| NH_4_Cl | 120 | Na2MoO_4_ ·2H_2_O | 0.39 |
| K2HPO_4_ ·3H_2_O | 19 | CuSO_4_ ·5H_2_O | 0.08 |
| MgSO_4_ ·7H_2_O | 50 | Co(NO3)_2_ ·6H_2_O | 0.05 |
| CaCl_2_ ·2H_2_O | 5 | — | — |

All statistical significance tests in the experiments were two-sided, and results were considered statistically significant when the P value and FDR q value were < 0.05. Error bars were defined as SD (n = 3, replicates). For operational parameters (temperature, pH, DO), samples were collected in triplicate at the same time each day and averaged. For water quality indicators, samples were collected from the middle portion of the effluent during three corresponding time points across three operational cycles. For biomass measurements, SVI, zeta potential, and particle size, samples were collected at the same time point over three consecutive days during the stable operation phase at the same time each day. For metagenomic analyses, four replicate samples were collected every other day at the same time point during the late stable phase of reactor operation when physicochemical parameters and effluent quality remained stable.

**SUPPLEMENTARY** **NOTE 5: Energy Consumption Calculation Based on Coarse-grained Method**

We can estimate of energetic costs to facilitate the biosystem comparison of chemotactic thresholds between cells, based on the developed methodology. To streamline the process, a coarse-grained approach is employed for cost accounting (S31, S32). Specific cellular processes incur associated energy expenditures, quantified using Gibbs free energy. We can estimate of energetic costs to facilitate the biosystem comparison of chemotactic thresholds between cells, based on the developed methodology. To streamline the process, a coarse-grained approach is employed for cost accounting (S31, S32). Specific cellular processes incur associated energy expenditures, quantified using Gibbs free energy. Specifically, within this system, the flagellar motion of cells necessitates approximately10^4-6^ ATP/s, whereas the cost for chemotaxis is about10^3-4^ ATP/s (S33), with diffusion constants calculated based on empirical values (S32, S33). This approach allows for the estimation of the time required to achieve an aggregated state and the associated costs. This is achieved through quantifying Gibbs free energy changes, an efficacious method for assessing the ATP equivalents expended per unit time during cellular activities. The energy cost of a cellular process can be estimated by computing the ATP equivalents consumed per intermediate step associated with the process per unit time.

$\text{t1≈}\text{X}^{2}\text{/D}$ (28)

$\text{D=}\frac{\text{K}_{\text{β}}\text{T}}{\text{6πηα}}$ (29)

$\text{AT}\text{P}_{\text{total}}\text{=AT}\text{P}_{\text{U}}\text{×t}$ (30)

$\text{t=ΔX/}\bar{\text{v}}$ (31)

$\bar{v}=\frac{\int_{-\infty}^{+\infty} xf(x)dx}{\Delta t}$ (32)

Here, D demotes Diffusion constant: t1 is diffusion effect delay time; and t represents movement time, and f (x) is probability distribution function of displacement density; kβ represents the Boltzmann constant.

In our analysis employing a cell counting method that excludes biomass growth considerations in the bacteriophyte system, we established a microbial concentration of 107 cells/mL within the biological system. Based on the reactor's effective working volume, we estimated a total cell count of 1.5×10^10^ cells. The diffusion time difference between the two reactor systems was negligible and therefore disregarded in our calculations. For individual cells, the ATP consumption rates for flagellar motion and chemotaxis ranged between 10^3^-10^6^ ATP/s, establishing our ATP unit (ATP_U) within this range. Using the reactor's characteristic dimensions as boundaries, we determined the mean chemotactic distance (ΔX) between bacterial cells and the average travel distance derived from probability density function (PDF) distribution analysis. The microalgae-bacteria granular system and bacterial granular system exhibited mean velocities of 60.18 μm/s and 39.02 μm/s respectively when approaching equilibrium positions based on the integrated model. These velocities corresponded to calculated time requirements of 3075.35 s and 1994.02 s, yielding a time difference of approximately 10^3^ s. Through corrected model simulations of equilibrium displacement distribution and mean velocity, we estimate the system achieves an energy expenditure reduction of 1.5×10^15-19^ ATP.

Quantifiable parameters facilitate the selection of more cost-effective chemotactic strategies, thereby enhancing directed ecological modulation at the scale of cell motility and improving the efficiency of operational response systems. The proportional relationship between ATP consumption reduced by flagellar motion and chemotaxis and the overall cellular energy flux enhances the energy allocation for other cellular processes, including biosynthesis, signaling, maintaining chemical gradients, error correction, motility, gene regulation, and building of cellular structures, such as the cytoskeleton.

SUPPLEMENTARY FIGURE 1: Simulation Framework and Reactor Operation


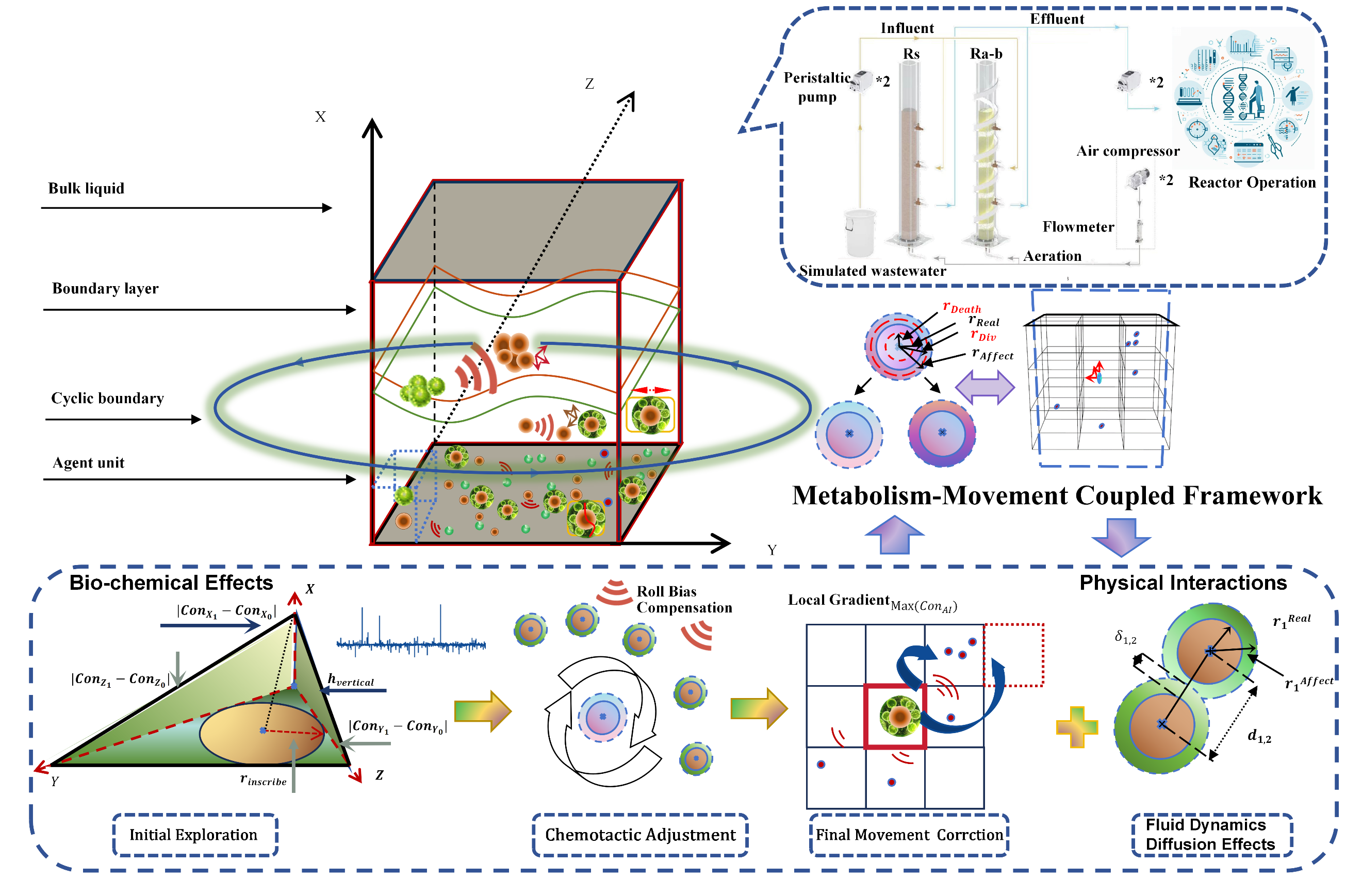


SUPPLEMENTARY FIGURE 2: Sensitivity Analysis


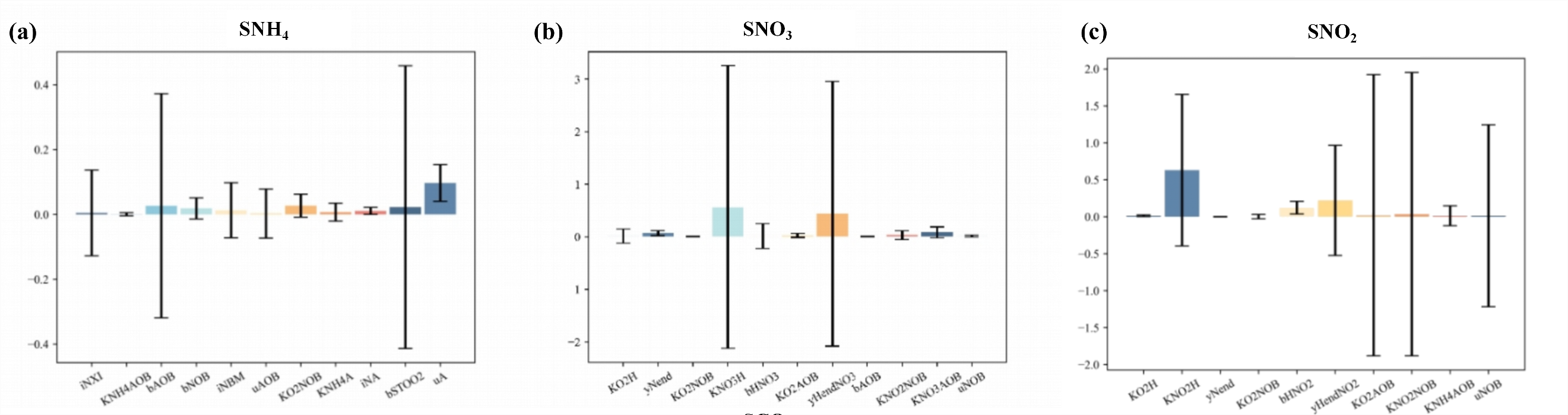


(a) sensitivity analysis outcomes for ammonia-nitrogen (SNH4), where the impact factors of maximum specific ammonia oxidizing bacteria growth rate (uA), decay coefficient of ammonia oxidizing bacteria (bAOB), and half-saturation constant for oxygen uptake by nitrite oxidizing bacteria (KO2NOB) exert considerable influence on SNH4, with respective sensitivity coefficients of 0.097, 0.027, and 0.027; (b) sensitivity analysis regarding nitrate-nitrogen (SNO3), highlighting that the half-saturation constant for nitrate uptake by heterotrophic bacteria (KNO3H), yield coefficient of heterotrophic bacteria using nitrate as an electron acceptor (yHendNO3), and half-saturation constant for nitrate uptake by ammonia oxidizing bacteria (KNO3AOB) are the parameters with the greatest impact on SNO3, with sensitivity coefficients of 0.564, 0.441, and 0.086, respectively. (c) sensitivity analysis for nitrite-nitrogen (SNO2), indicating that the half-saturation constant for nitrite uptake by heterotrophic bacteria (KNO2H), yield coefficient of heterotrophic bacteria using nitrite as an electron acceptor (yHendNO2), and decay coefficient of heterotrophic bacteria utilizing nitrite (bHNO2) have a substantial effect on SNO2, with sensitivity coefficients of 0.63, 0.221, and 0.122, respectively.

SUPPLEMENTARY FIGURE 3: Cell movement trajectory of Rs and Ra-b


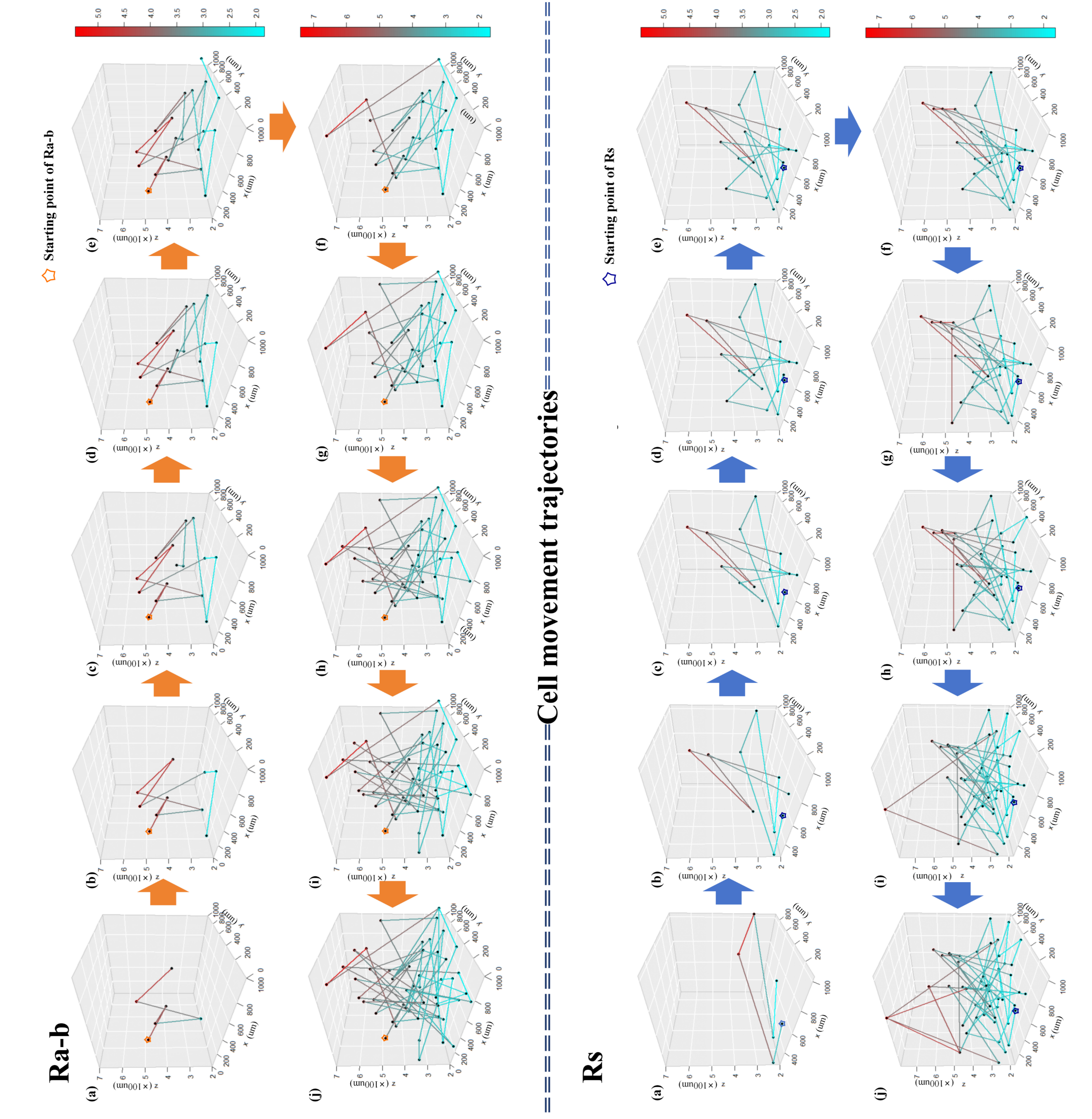


Cell movement trajectory of Ra-b and Rs,the color bar correspond to the movement height.

SUPPLEMENTARY FIGURE 4: Cell movement trajectory in two-dimension of Rs and Ra-b


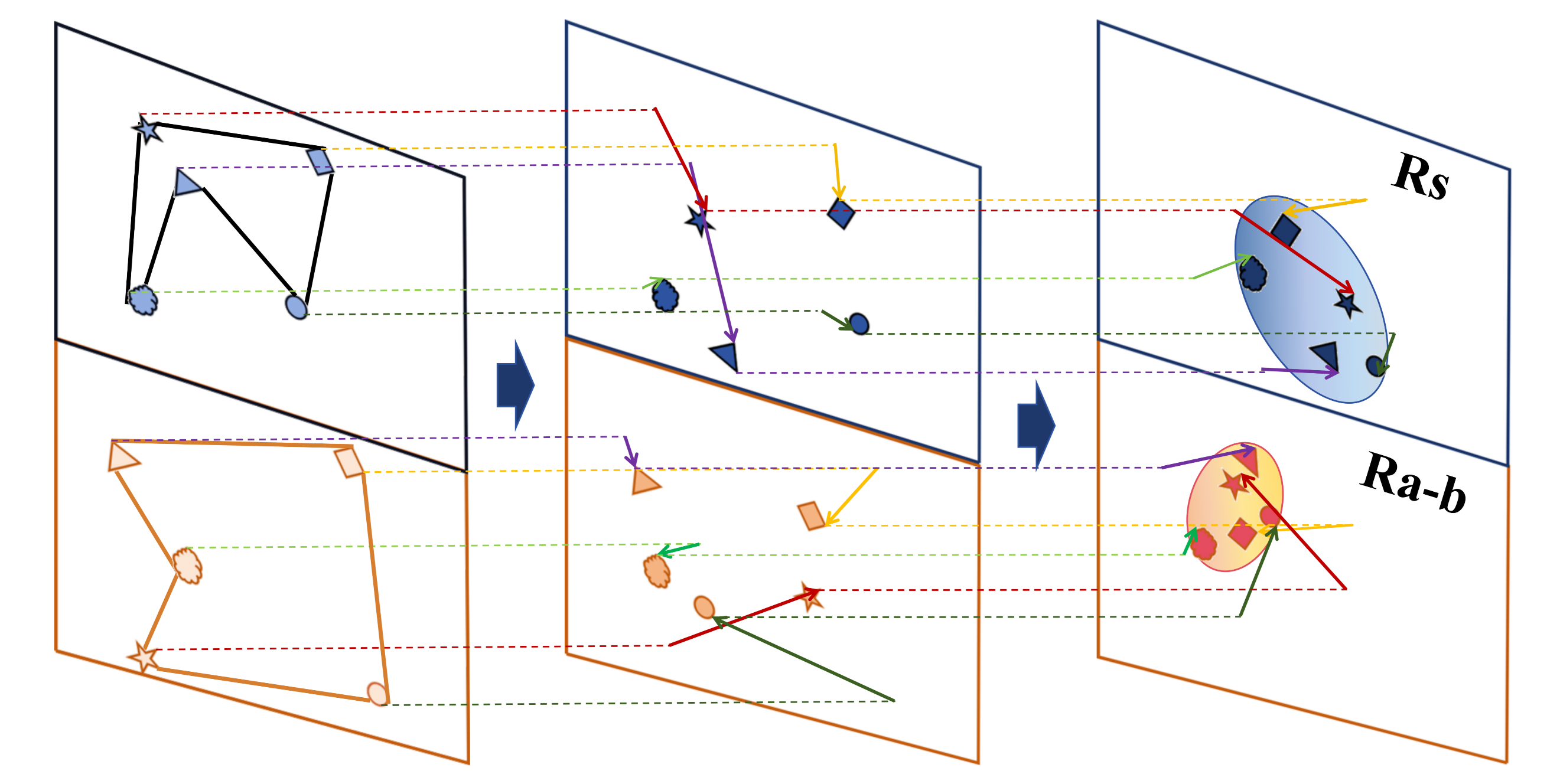


Simulation of the positions of multiple bacterial cells in motion, where the connecting lines of the projected arrows represent the travel distance. The figure clearly shows that the microalgae-bacteria system displays greater movement displacement.

SUPPLEMENTARY FIGURE 5：Dynamic simulation of microalgae and bacteria


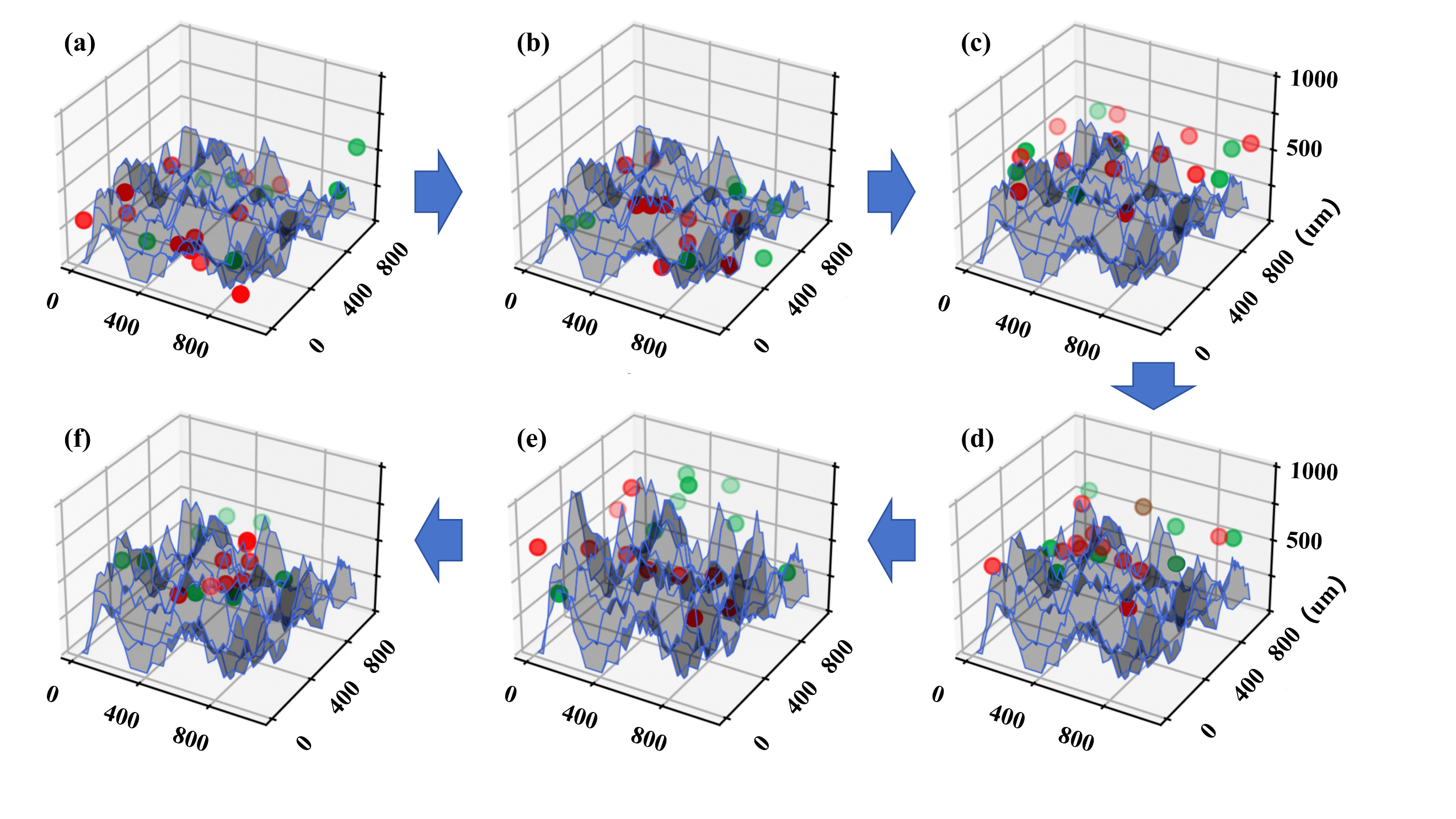


Dynamic simulation of microalgae and bacterial clusters movement in Ra-b

SUPPLEMENTARY FIGURE 6: Signal molecule concentrations analysis of the Rs and Ra-b systems


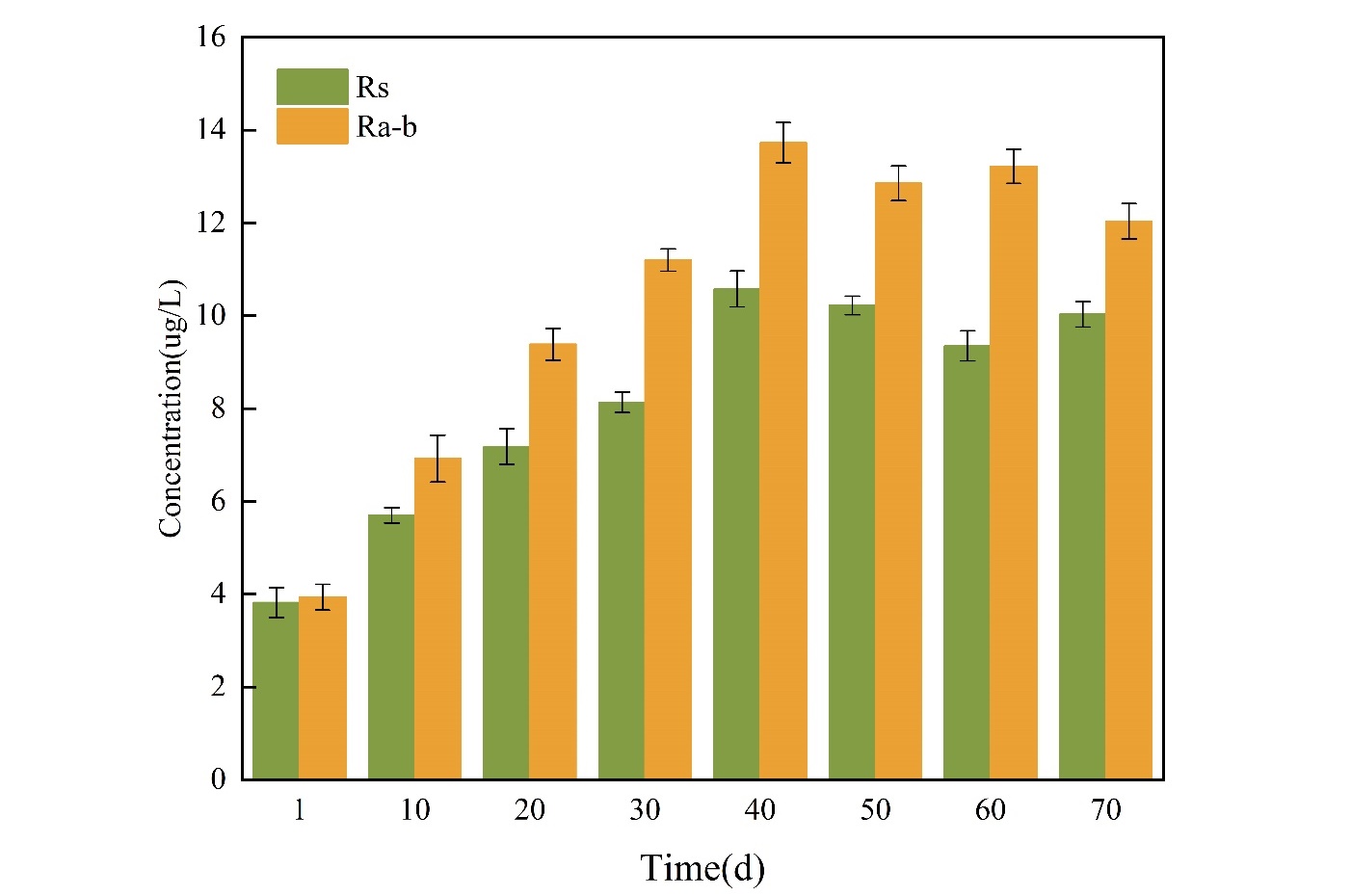


SUPPLEMENTARY FIGURE 7: Heat map of key metabolic pathways in two system


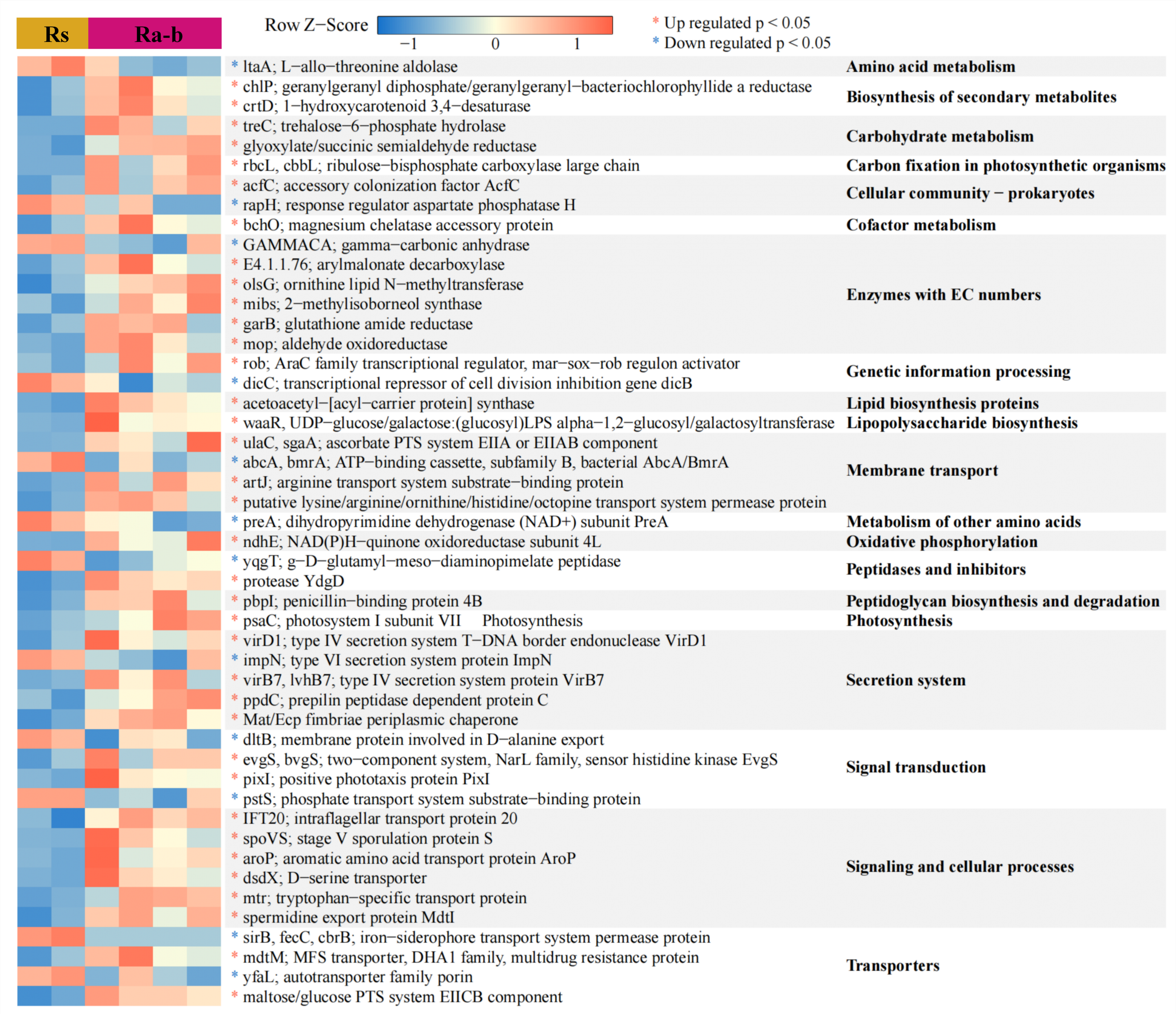


Red and blue * indicate a significantly up-regulated and down-regulated system stability, substrate utilization-related metabolic potential, respectively (p <0.05)

SUPPLEMENTARY FIGURE 8: A comparative genomic investigation of the metabolic pathways implicated in bacterial motility


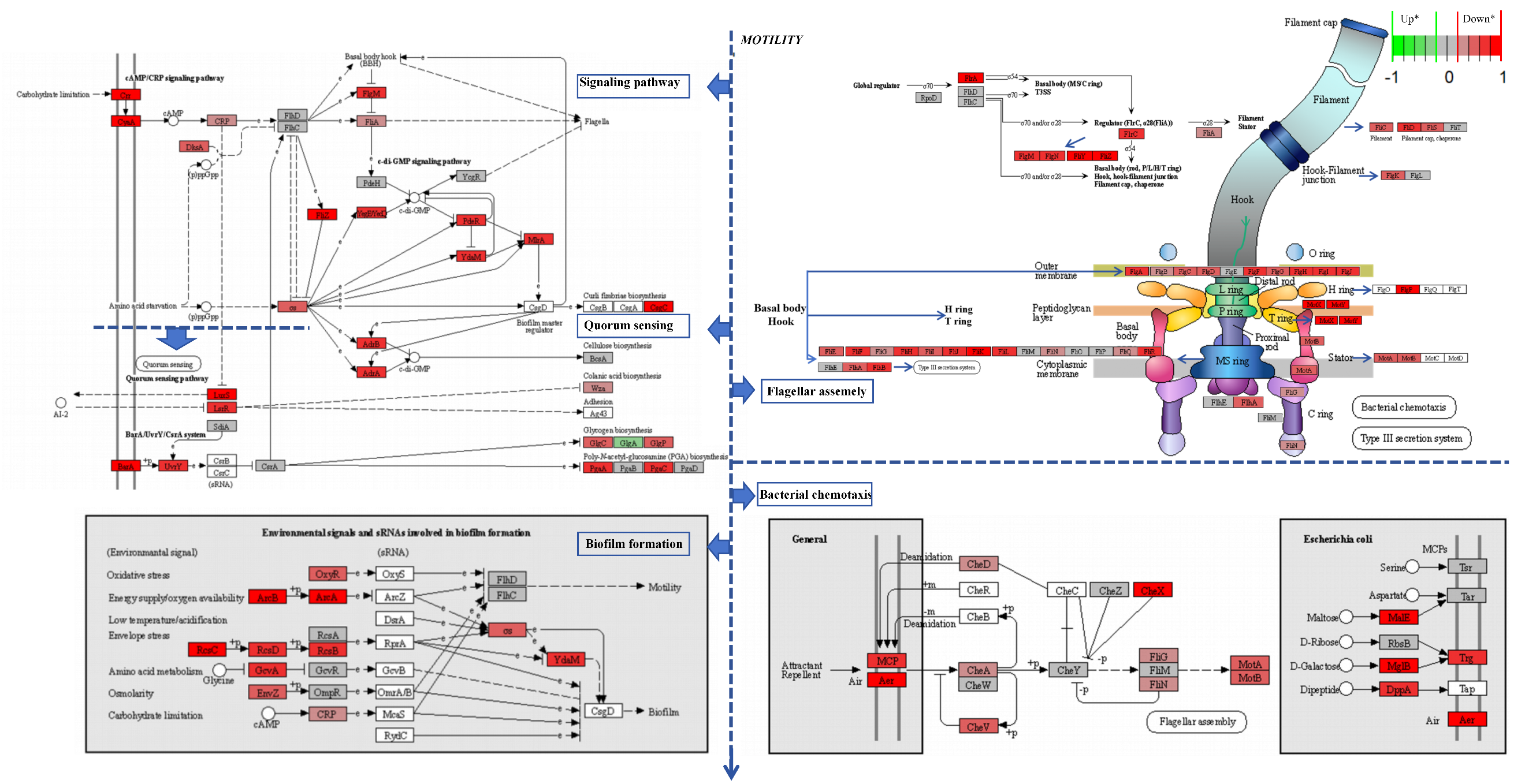


A comparative genomic investigation of the metabolic pathways implicated in bacterial motility reveals a substantial up-regulation, which indicated in red, and down-regulation, which indicated in green, of pertinent genes within the microalgae-bacteria system (p < 0.05). Significantly up-regulated genes, highlighted in red, include those governing motility, quorum sensing, and the bacteria chemotaxis, collectively suggesting an augmented propensity for bacterial motility within the microalgae-bacteria consortium.

SUPPLEMENTARY FIGURE 9: Pollutant Removal Efficiency


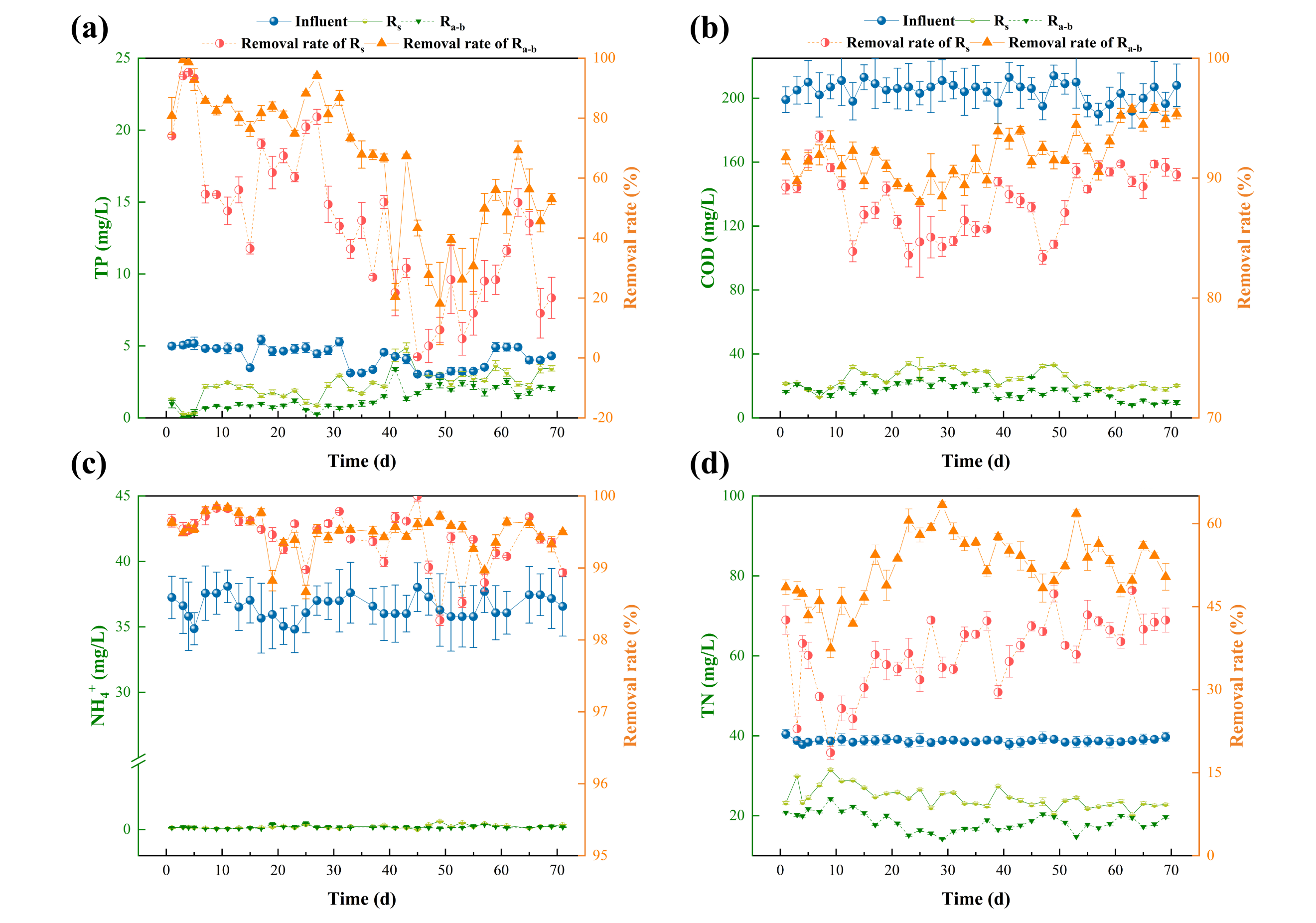


SUPPLEMENTARY FIGURE 10: Model Performance on Ra-b and Rs systems


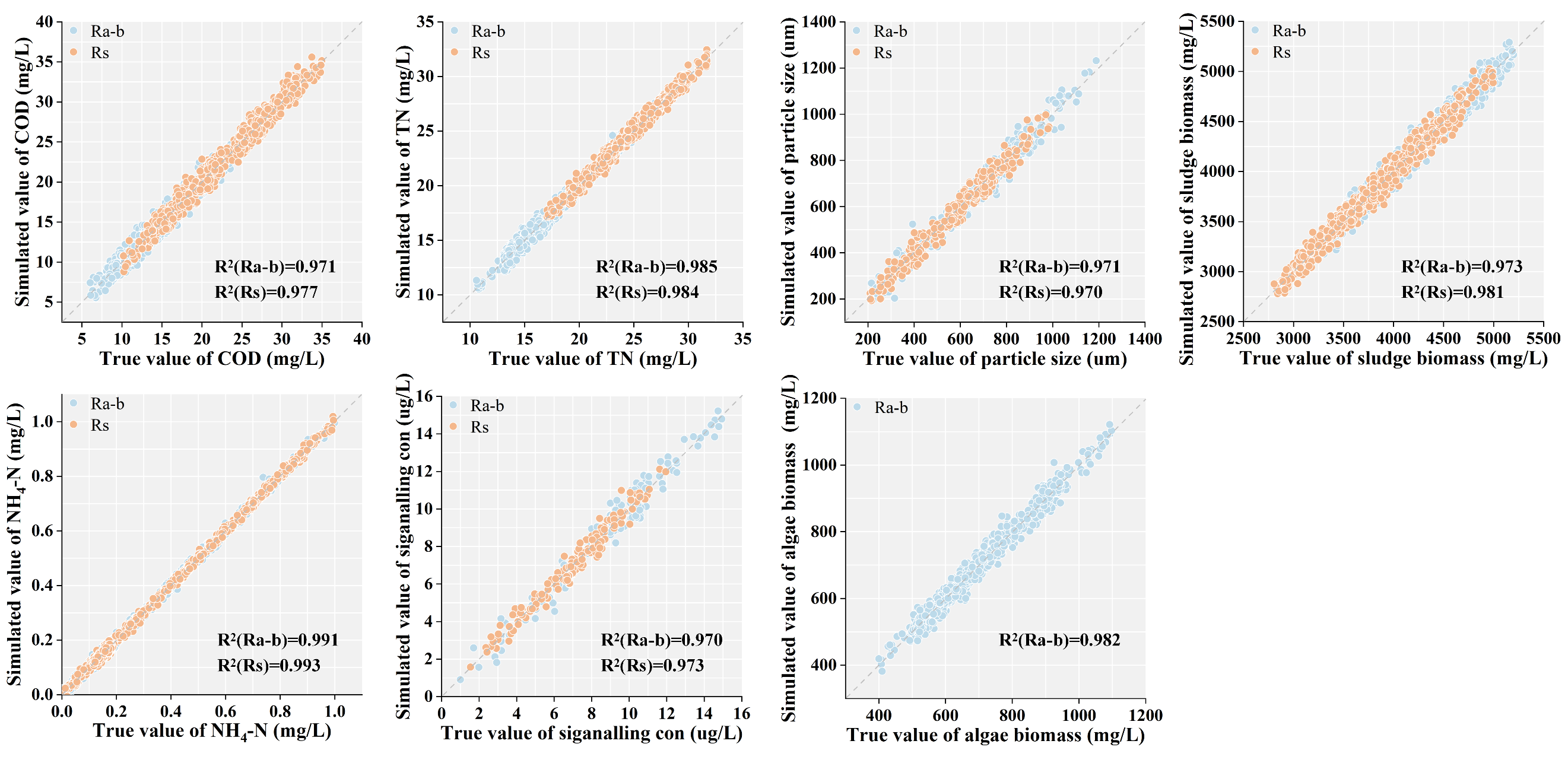


**Reference:**

[S1] Reichert, P., Borchardt, D., Henze, M., Rauch, W., Shanahan, P., Somlyódy, L., Vanrolleghem, P. 2001. River water quality model no. 1 (RWQM1): II. Biochemical process equations. Water Science and Technology, 43(5), 11-30.

[S2] Zhu, A., Guo, J., Ni, B.-J., Wang, S., Yang, Q., Peng, Y. 2015. A novel protocol for model calibration in biological wastewater treatment. Scientific reports, 5, 8493.

[S3] G. Sin, A. Guisasola, D.J. De Pauw, J.A. Baeza, J. Carrera, P.A. Vanrolleghem, A new approach for modelling simultaneous storage and growth processes for activated sludge systems under aerobic conditions, Biotechnology and Bioengineering, 92 (2005) 600-613.

[S4] Wiesmann, U. 1994. Biological nitrogen removal from wastewater. Biotechnics/wastewater, 113-154.

[S5] Manser, R., Gujer, W., Siegrist, H. 2005. Consequences of mass transfer effects on the kinetics of nitrifiers. Water Research, 39(19), 4633-4642.

[S6] Sheng, Huajun;Ni, Shenzhou;Wang, Yuyin;Yuan, Rui;Su, Kuizu;Hao, Tianwei.Uncertainty and sensitivity analysis of algal-bacterial model under different ranges of parameter variation.[J].Biochemical Engineering Journal,2022,Vol.179: 108334

[S7] Henze, M., Gujer, W., Mino, T., van Loosdrecht, M.C. 2000. Activated sludge models ASM1, ASM2, ASM2d and ASM3. IWA publishing.

[S8] Iacopozzi, I., Innocenti, V., Marsili-Libelli, S., Giusti, E. 2007. A modified Activated Sludge Model No. 3 (ASM3) with two-step nitrification–denitrification. Environmental Modelling & Software, 22(6), 847-861.

[S9] Arashiro, L.T., Rada-Ariza, A.M., Wang, M., Van Der Steen, P., Ergas, S.J. 2017. Modelling shortcut nitrogen removal from wastewater using an algal–bacterial consortium. Water Science and Technology, 75(4), 782-792.

[S10] Koch, G., et al. "Calibration and validation of activated sludge model no. 3 for Swiss municipal wastewater." Water Research 34.14 (2000): 3580-3590.

[S11] Kaelin, D., Manser, R., Rieger, L., Eugster, J., Rottermann, K., Siegrist, H. 2009. Extension of ASM3 for two-step nitrification and denitrification and its calibration and validation with batch tests and pilot scale data. Water Research, 43(6), 1680-1692.

[S12] Wu, Y.-H., Li, X., Yu, Y., Hu, H.-Y., Zhang, T.-Y., Li, F.-M. 2013. An integrated microalgal growth model and its application to optimize the biomass production of Scenedesmus sp. LX1 in open pond under the nutrient level of domestic secondary effluent. Bioresource technology, 144, 445-451.

[S13] Novak, J.T., Brune, D.E. 1985. Inorganic carbon limited growth kinetics of some freshwater algae. Water research, 19(2), 215-225.

[S14] Solimeno, A., Parker, L., Lundquist, T., García, J. 2017b. Integral microalgae-bacteria model (BIO_ALGAE): application to wastewater high rate algal ponds. Science of the Total Environment, 601, 646-657.

[S15] M. Henze, W. Gujer, T. Mino, M.C. van Loosdrecht, Activated sludge models ASM1, ASM2, ASM2d and ASM3, IWA publishing, 2000.

[S16] A. Solimeno, R. Samsó, E. Uggetti, B. Sialve, J.-P. Steyer, A. Gabarró, J. García, New mechanistic model to simulate microalgae growth, Algal Research, 12 (2015) 350-358.

[S17] D. Kaelin, R. Manser, L. Rieger, J. Eugster, K. Rottermann, H. Siegrist, Extension of ASM3 for two-step nitrification and denitrification and its calibration and validation with batch tests and pilot scale data, Water Research 43(2009) 1680–1692

[S18] Siderius D W. 3 Engineering Statistics[J]. CHEMICAL ENGINEERING HANDBOOK, 199.

[S19] Metzler R, Klafter J. The random walk's guide to anomalous diffusion: a fractional dynamics approach[J]. Physics reports, 2000, 339(1): 1-77.

[S20] He X, Zhu Y, Epstein A, et al. Statistical variances of diffusional properties from ab initio molecular dynamics simulations[J]. npj Computational Materials, 2018, 4(1): 18.

[S21] Grassberger P. Grassberger-procaccia algorithm[J]. Scholarpedia, 2007, 2(5): 3043.

[S22] Grassberger P, Procaccia I. Characterization of strange attractors[J]. Physical review letters, 1983, 50(5): 346.

[S23] Grassberger P, Procaccia I. Measuring the strangeness of strange attractors[J]. Physica D: nonlinear phenomena, 1983, 9(1-2): 189-208.

[S24] Dickinson R B, Tranquillo R T. Optimal estimation of cell movement indices from the statistical analysis of cell tracking data[J]. AIChE Journal, 1993, 39(12): 1995-2010.

[S25] Xu X, Cui D, Li Y, et al. Research on ship trajectory extraction based on multi-attribute DBSCAN optimisation algorithm[J]. Polish Maritime Research, 2021, 28(1): 136-148.

[S26] Dellino G, Meloni C. Uncertainty management in simulation-optimization of complex systems[M]. Boston, MA, USA: Springer, 2015.

[S27] Zhang, X‐Y., et al. "Sobol sensitivity analysis: a tool to guide the development and evaluation of systems pharmacology models." CPT: pharmacometrics & systems pharmacology 4.2 (2015): 69-79.

[S28] Lilburne, Linda, and Stefano Tarantola. "Sensitivity analysis of spatial models." International Journal of Geographical Information Science 23.2 (2009): 151-168.

[S29] Saltelli, Andrea. "Making best use of model evaluations to compute sensitivity indices." Computer physics communications 145.2 (2002): 280-297.

[S30] Saltelli, Andrea, et al. "Variance based sensitivity analysis of model output. Design and estimator for the total sensitivity index." Computer physics communications 181.2 (2010): 259-270.

[S31] Keegstra J M, Carrara F, Stocker R. The ecological roles of bacterial chemotaxis[J]. Nature Reviews Microbiology, 2022, 20(8): 491-504.

[S32] Yang X, Heinemann M, Howard J, et al. Physical bioenergetics: Energy fluxes, budgets, and constraints in cells[J]. Proceedings of the National Academy of Sciences, 2021, 118(26): e2026786118.

[S33] Milo R, Phillips R. Cell biology by the numbers[M]. Garland Science, 2015.
